# mTORC1 Activation in *Chx10*-Specific *Tsc1* Knockout Mice Accelerates Retina Aging and Degeneration

**DOI:** 10.1155/2021/6715758

**Published:** 2021-11-05

**Authors:** Yu-Qing Rao, Yu-Tong Zhou, Wenchuan Zhou, Jia-Kai Li, Baojie Li, Jing Li

**Affiliations:** ^1^Department of Ophthalmology, Xinhua Hospital Affiliated to Shanghai Jiao Tong University School of Medicine, Shanghai, China; ^2^Bio-X Institutes, Key Laboratory for the Genetics of Developmental and Neuropsychiatric Disorders, Ministry of Education, Shanghai Jiao Tong University, Shanghai, China

## Abstract

Age-associated decline in retina function is largely responsible for the irreversible vision deterioration in the elderly population. It is also an important risk factor for the development of degenerative and angiogenic diseases. However, the molecular mechanisms involved in the process of aging in the retina remain largely elusive. This study investigated the role of mTORC1 signaling in aging of the retina. We showed that mTORC1 was activated in old-aged retina, particularly in the ganglion cells. The role of mTORC1 activation was further investigated in *Chx10-Cre;Tsc1^fx/fx^* mouse (*Tsc1*-cKO). Activation of mTORC1 was found in bipolar and some of the ganglion and amacrine cells in the adult *Tsc1*-cKO retina. Bipolar cell hypertrophy and Müller gliosis were observed in *Tsc1*-cKO since 6 weeks of age. The abnormal endings of bipolar cell dendritic tips at the outer nuclear layer resembled that of the old-aged mice. Microglial cell activation became evident in 6-week-old *Tsc1*-cKO. At 5 months, the *Tsc1*-cKO mice exhibited advanced features of old-aged retina, including the expression of p16^Ink4a^ and p21, expression of SA-*β*-gal in ganglion cells, decreased photoreceptor cell numbers, decreased electroretinogram responses, increased oxidative stress, microglial cell activation, and increased expression of immune and inflammatory genes. Inhibition of microglial cells by minocycline partially prevented photoreceptor cell loss and restored the electroretinogram responses. Collectively, our study showed that the activation of mTORC1 signaling accelerated aging of the retina by both cell autonomous and nonautonomous mechanisms. Our study also highlighted the role of microglia cells in driving the decline in retina function.

## 1. Introduction

Visual functions, including visual acuity, visual field sensitivity, contrast sensitivity, and dark adaptation threshold, deteriorate with age [1]. The age-related changes of the retina included the loss of retinal neurons, declined electroretinography (ERG) responses, increased oxidative stress, activation of microglial cells, and increased inflammatory responses. Aging is also an important risk factor for the development of degenerative and angiogenic retinal diseases, such as age-related macular degeneration, glaucomatous retinopathy, and diabetic retinopathy [2]. In humans, age-related thinning of inner retina, including the ganglion cell layer (GCL), inner plexiform layer (IPL), and inner nuclear layer (INL), the loss of retinal ganglion cells (RGCs), bipolar cells, and rod photoreceptors, and increased expression of senescence-related proteins have been reported [1, 3, 4]. However, the mechanisms which regulate the aging process of retina (retina aging) are yet to be elucidated.

Mammalian target of rapamycin complex 1 (mTORC1) is a protein complex that senses and integrates environmental and intracellular nutrient, growth factors, energy, and redox status and regulates protein synthesis [5]. mTORC1 signaling pathway is involved in the regulation of cell growth, proliferation, apoptosis, and inflammatory responses. In cells, the tuberous sclerosis complex, a protein complex consisting of TSC1 and TSC2, is a major upstream inhibitor of mTORC1 [6]. Deletion of *Tsc1* gene leads to the activation of mTORC1 in affected cells. On the other hand, rapamycin effectively inhibits mTORC1 therefore attenuates mTORC1-mediated signaling activity. The two best-characterized downstream effector molecules of mTORC1 are ribosomal protein S6 kinase (S6K) and eukaryotic translation initiation factor 4E- (eIF4E-) binding protein 1 (4E-BP1) [7]. mTORC1 phosphorylates and activates ribosomal protein S6 kinase (S6K), which subsequently phosphorylates ribosomal protein S6 (S6). Phosphorylation of 4E-BP1 by mTORC1 releases its binding from eIF4E, enabling it to form an active protein translation initiation complex and initiate cap-dependent protein translation.

Chx10 is a transcriptional factor involved in retinal development and bipolar cell differentiation [8]. The expression of *Chx10* is restricted in the anterior part of the optic vesicle during retina genesis and is gradually restricted to bipolar cells in adult mice [9]. Using *Chx10-cre* driven *Tsc1* conditional knockout mice (*Tsc1*-cKO), Choi et al. found that the activation of mTORC1 in *Chx10*-expressing cells shortened retinal progenitor cell cycle and subsequently accelerated progenitor cell differentiation and retina development [10]. Depending on the timing of *Tsc-1* knockout during retinal development, the activation of mTORC1 could also lead to the manifestation of ocular tuberous sclerosis complex [11]. Reduced mTORC1 signaling led to proliferation defect and excessive production of retinal ganglion cells [11]. mTORC1 is also expressed abundantly in adult retina, at a level higher than metabolically active brain and liver, especially in cells of the inner retina [12]. In ganglion cells, mTORC1 activity was needed for axonal survival after optic nerve injury [13–17]. However, Müller glial predominantly express mTORC2 rather than mTORC1 [12]. In photoreceptor cells, augmentation of mTORC1 activity extended the survival of cone cells in mouse with retinitis pigmentosa, possibly because it ameliorated the nutrition deprivation condition caused by rod cell death [18–20].

mTORC1 is also involved in the regulation of longevity and aging. Inhibition of mTORC1 signaling by rapamycin extended the longevity of all livings tested so far, from single cell yeast to nonhuman primates [21]. In human retinal pigment epithelial cells, the mTOR pathway showed age-associated changes [22]. In the OXYS rat, a rat model of accelerated aging due to overproduction of free radicals, rapamycin treatment ameliorated the incidence and severity of retinal degeneration [23]. However, it is not known whether mTORC1 signaling played a direct role in retina aging and neural retinal cell senescence. In this study, we showed that mTORC1 signaling was activated in old-aged retina. We found that ganglion cells of the old-aged retina had increased mTORC1 activity and the expression of senescence associated-*β*-galactosidase (SA-*β*-gal). To further study the effect of mTORC1 activation on retina aging, we used the adult *Tsc1*-cKO mice and found that the ablation of *Tsc1* in *Chx10*-expressing cells accelerated retinal aging as evidenced by progressively declined ERG responses, the elevation of oxidative stress level, the activation of microglial cells, the expression of senescence-related proteins, and a gene expression profile with features of aging retina. Furthermore, we found that the inhibition of microglial cells by minocycline prevented photoreceptor cells loss and partially restored the ERG responses. Collectively, our study demonstrated mTORC1 activation accelerated retina aging and retinal degeneration. Our results also suggested that microglial cell activation is an important factor which lead to overall functional deterioration in the retina.

## 2. Materials and Methods

### 2.1. Animal Husbandry and Genotyping

*Chx10-Cre* (*Tg(Chx10-EGFP/cre,-ALPP)2Clc/J*, *cat. 005105*), *Tsc1-flox* (*Tsc1^tm1Djk^/J*, cat. 005680), and the tdTomato reporter mice (*B6.Cg-Gt(ROSA)26Sortm9(CAG-tdTomato)Hze/J*, cat. 007909) were obtained from the Jackson Laboratory and housed in a specific-pathogen-free (SPF) mouse facility under 12-hour light/dark cycle with unrestricted access to food and water in Xinhua Hospital Affiliated to Shanghai Jiao Tong University School of Medicine. They were mated to produce the *Chx10-Cre;Tsc1^f/f^* mice (*Tsc1*-cKO). Unless otherwise specified, the control mice used in this study were *Chx10-Cre;Tsc^+/+^*. All procedures involving animals were in compliance with the ARVO statement for the Use of Animals in Ophthalmic and Vision Research and approved by the Institutional Animal Care and Use Committee of Xinhua Hospital.

Animals were genotyped by PCR analysis using genomic DNA extracted from tail biopsies. Cre recombinase DNA was detected using Cre-F (5′-TTTCCCGCAGAACCTGAAGA-3′) and Cre-R (5′-GGTGCTAACCAGCGTTTTCGT-3′). A 430 basepair (bp) amplicon was expected. The floxed *Tsc1* was detected using IMR4008 (5′-GTCACGACCGTAGGAGAAGC-3′) and IMR4009 (5′-GAATCAACCCCACAGAGCAT-3′). The floxP containing DNA yielded a 230 bp product, and the wildtype DNA yielded a 193 bp product.

### 2.2. Histological and Immunofluorescent Microscopy

Animals were given a lethal dose of sodium pentobarbital (120 mg/kg body weight) and were either enucleated or perfused immediately with fresh-made 4% paraformaldehyde (PFA). The retina cup free of vitreous and retinal pigment epithelium was fixed in PFA overnight at 4°C, followed by sequential dehydration in 10%, 20%, and 30% sucrose, embedded in optimal cutting temperature compound (OCT), and sectioned at 10 *μ*m thickness using a Leica microtome (CM1950, Leica Biosystems, Wetzlar, Germany).

Hematoxylin and eosin (H&E) staining was performed according to the standard protocol and visualized under light microscope. For immunofluorescent staining, the tissue sections were permeabilized with 0.5% Triton X-100 for 60 mins, blocked with 10% goat serum and 0.1% Triton X-100 in PBS at room temperature, and incubated with desired primary antibody at 4°C overnight. The antibodies used in the present study (including those for Western blot analysis below) are listed in Supplementary Table S1. The slides were visualized under fluorescent microscope (Leica DMi3000B) or confocal microscope (Leica TCS SP8).

### 2.3. Senescence-Associated *β*-Galactosidase Staining

A senescence-associated *β*-galactosidase (SA-*β*-Gal) staining kit (Catalog no. 9860; Cell Signaling Technology, Danvers, MA, USA) was used according to the manufacturer's instruction. Briefly, frozen sections of mouse retina were restored to room temperature, fixed in the provided fixative for 15 mins, washed with PBS 3 times, and then incubated in freshly prepared staining solution for 12 hrs at 37°C. Pictures were taken under light microscope. The original blue stained images were converted to grayscales to increase the contrast.

### 2.4. Western Blot Analysis

Freshly isolated retina cup was lysed in RIPA buffer (Cat. 9806S, Cell Signaling Technology) containing 1 mM PMSF and 1x protease inhibitor cocktail (cOmplete Protease Inhibitor Cocktail Tablets, Cat. 5892970001, Roche Diagnostics, Basel, Switzerland) with sonication. Protein concentration was measured using Micro BCA protein assay kit (Cat. 23235, Thermo Fisher Scientific, Shanghai, China), and 30 *μ*g of total tissue lysates was loaded onto SDS-PAGE, transferred to nitrocellulose membrane (1620115, Bio-Rad Laboratories, Hercules, CA, USA), and probed with indicated antibodies (Supplementary Table S1). Proteins were visualized using chemiluminescent HRP substrate (Immobilon Western, WBKLS0500, MilliporeSigma, Shanghai, China), and the signals were captured by Bio-Rad Gel Doc XR+ system. For quantitative analysis, the densitometrical value of the protein bands was analyzed with ImageJ and normalized to *β*-actin.

### 2.5. RNA Extraction and Real-Time PCR

Total RNA was extracted using Trizol reagent (Thermo Fisher Scientific, Shanghai, China) and the RNA Clean & Concentrator-5 kit (R1013, Zymo Research, Irvine, CA, USA). Complementary DNA was synthesized using PrimeScript RT Reagent Kit (RR037A, Takara, Japan). Real-time PCR analysis was performed using TB Green Premix Ex Taq II reagent (RR820A, Takara, Japan). Gene expression was compared by *δ*Ct method using *Actb* as internal control. Primer sequences were listed in Supplementary Table S2.

### 2.6. Electroretinograms

The Espion E3 console in conjunction with the Color Dome (Diagnosys LLC, Lowell, MA, USA) was used for Ganzfeld ERG recording. The procedure was adopted from a detailed description for mouse ERG examination [24]. Briefly, mice were weighted, tagged, and dark-adapted overnight before the experiment. Sodium pentobarbital was used as anesthesia at the dose of 80 *μ*g/g body weight and injected intraperitoneally in the dark. Once the mouse was anesthetized, it was gently moved to a warm metal plate to maintain the body temperature at 37°C. The pupil was dilated with 0.5% tropicamide-0.5% phenylephrine (Mydrin-P, Santen Pharmaceutical Co., Ltd., Japan) about 10 mins before the recording. A thin layer of ofloxacin eye ointment (Sinqi Pharmaceutical, Shenyang, China) was applied to the cornea surface which served as a preventative medication for infection and as a lubricant. Platinum wire ring electrodes were positioned on the surface of both cornea for binocular ERG recordings. A reference electrode was placed subcutaneously at the middle of the forehead region. A ground electrode was placed at the back near the tail.

For the dark-adapted single-flash ERG recording, the mouse was placed in the dome with no background illumination. Single white-flash stimuli at -4.3, -4, -3, -2, -1.5, -1, -0.5, 0, 0.4, 1, and 1.3 log cd∙s/m^2^ were given at the interstimulus intervals of 40 s. Band-pass filtering was applied from 0.3 to 300 Hz. For each stimulus, five responses were recorded and averaged. For the light-adapted ERG recording, the mouse was light adapted for 10 mins at a static background light of 1.3 log cd/m^2^. White-flash stimuli at -2, -1.5, -1, -0.5, 0, 0.5, 1, and 1.5 log cd∙s/m^2^ were given at the interstimulus intervals of 10 secs. For each stimulus, ten responses were taken and averaged.

The total recording time was 420 ms, including a 20 ms preflash recording time. The amplitude of a-wave was measured from the prestimulus baseline to the most negative trough of the ERG. The amplitude of the b-wave was measured from the trough of the a-wave to the most positive peak of the ERG which followed the a-wave. Peak times were measured from flash onset to the peak of a- and b-waves.

### 2.7. Microarray Analysis of Gene Expression

Three 5-month-old *Tsc1*-cKO mice, three age-matched control, two 24-month-old C57BL/6, and two 5-month-old *Tsc1*-cKO mice after rapamycin treatment were subjected to gene expression analysis. The Agilent SurePrint G3 Mouse Gene Expression v2 8x60K microarray (Design ID: 074809) was used. The array contained 56605 probes which covered 27307 genes. Total RNA was quantified by NanoDrop ND-2000, and the integrity of RNA was assessed using Agilent Bioanalyzer 2100. Sample labeling, microarray hybridization, and washing were performed according to the manufacturer's suggestions. Agilent Scanner G2505C was used to scan the arrays at the end.

The Feature Extraction software (version 10.7.1.1, Agilent Technologies) was used to analyze array images to get raw data. It was then normalized with the quantile algorithm. The probes that were flagged as “detected” in at least 1 out of 2 conditions were chosen for further analysis. For each expressed gene, the fold change between groups and *p* value was calculated. Differentially expressed genes (DEGs) were identified if the fold change was 2 or more and the *p* value equaled or less than 0.05. Afterwards, Gene Set Enrichment Analysis (GSEA) and Gene Ontology (GO) analysis were performed to explore the collective functions of the DEGs. GSEA was conducted based on the normalized gene expression values from microarray data according to the methods described previously [25]. The gene sets analyzed were acquired from the GO database. A gene set-based permutation test of 1000 permutations was applied, and genes were ranked according to the signal2noise method. All other parameters were set to GSEA defaults. GO enrichment analysis of differentially expressed genes was performed using R based on the hypergeometric distribution.

### 2.8. Dihydroethidium (DHE) Fluorescent Staining

DHE fluorescent staining was performed to determine the reactive oxygen species (ROS) level in the retina *in vivo* [26, 27]. Briefly, freshly made DHE (10 mg/mL in anhydrous dimethylsulfoxide mixed with equal volume of 2x sterile PBS) was injected intraperitoneally at the dose of 50 mg/kg body weight. The mice were sacrificed 2 hrs after the injection, and the eyes were enucleated, embedded in OCT, and cryo-sectioned at a thickness of 10 *μ*m. The slides were then washed twice in PBS and mounted with ProlongGold anti-fade reagent (P10144, Thermo Fisher Scientific) and scanned using a 532 nm excitation laser and a 580 nm long-pass detection filter. The intensity of the fluorescence of the entire section was quantified using ImageJ and used for comparison between groups.

### 2.9. Lipid Peroxidation Analysis

The level of lipid peroxidation was indicated by measuring the concentration of malondialdehyde (MDA) in the retina (ab118970, Abcam, Shanghai, China) according to the manufacturer's instruction. Briefly, retina tissue was homogenized in 300 *μ*L MDA lysis buffer supplemented with lipid peroxidation inhibitor. After centrifugation, 10 *μ*L of the supernatant was used for protein quantification, and 200 *μ*L of the supernatant was used to determine the amount of MDA based on the reaction with thiobarbituric acid (TBA) in a 96-well microplate. The final product of MDA-TBA adduct was quantified colorimetrically on a microplate reader at 532 nm.

### 2.10. Rapamycin Treatment

Rapamycin (HY-10219, MedChemExpress) was dissolved in 100% alcohol at the concentration of 25 mg/mL and further diluted to 1 mg/mL using a water-based solvent containing 5% Tween-80 and 5% PEG-400. The experimental mice were given intraperitoneal injections of 5 *μ*g/g body weight every day for 30 days and sacrificed 3 days after the last injection [28]. The control group received solvent at the same time as the experimental group.

### 2.11. Minocycline Treatment

Minocycline hydrochloride (HY-17412, MedChemExpress, Shanghai, China) at the dose of 50 mg/kg was injected intraperitoneally to mice daily for 1 month as described [29, 30].

### 2.12. Statistical Analysis

The Statistical Package for the Social Sciences (*SPSS*) version 21 was used for data analysis. Unless otherwise specified, the average and standard error (SE) of data from repeated experiments were presented. Unpaired Students' *t*-test or two-way analysis of variance (ANOVA) was used to determine the statistical significance between two groups. A *p* value that equals or less than 0.05 was considered as statistically significant.

## 3. Results

### 3.1. mTORC1 Activity Is Increased in Retina of Old-Aged C57BL/6 Mice

**Figure 1:**
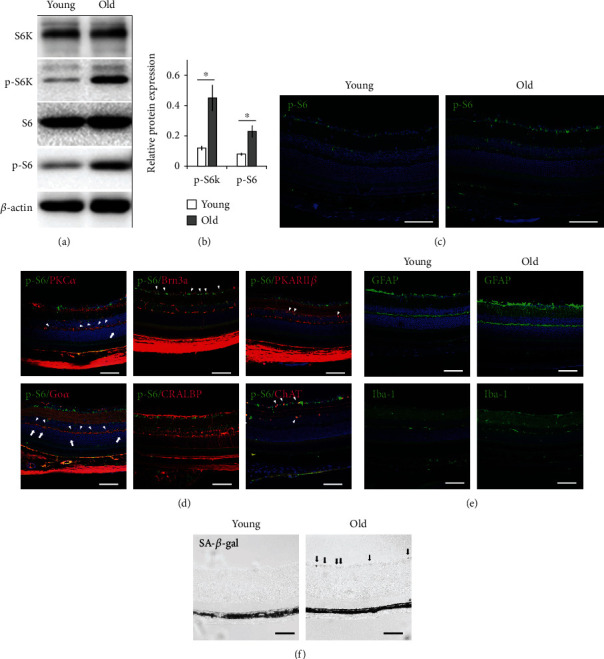
Increased mTORC1 activity in 24-month-old C57BL/6 retina corresponded with cell senescence. (a) Representative Western blot analysis of total ribosomal protein S6 (S6), phospho-S6 (p-S6), total S6 kinase (S6K), and phospho-S6 kinase (p-S6K) in retinal extracts of 24- (old) and 3-month-old (young) mice. The experiments were repeated three times with one pair of samples at each time. (b) Relative expression of phosphorylated S6 and S6K proteins in control and 24-month-old C57BL/6 retina. Phosphorylation status of each protein was calculated by dividing the intensity of the band of the phosphorylated protein by the intensity of the band of the corresponding total protein. The number from each experiment was averaged, and the error bars represented SE. ^∗^*p* < 0.05 by unpaired Student's *t*-test between 24- and 3-month-old groups. (c) Representative immunofluorescent staining of p-S6 on 24- (old) and 3-month-old (young) mouse retina. (d) Costaining of p-S6 with markers for various retinal cells indicated the activation of mTORC1 in bipolar cells (PKC*α*, Go*α*, PKARII*β*), ganglion cells (Brn3a), and amacrine cells (ChAT) but not in Müller glial cells (CRALBP) (arrowheads) of 24-month-old C57BL/6 mouse retina. Arrows indicated aberrant dendritic tip endings of bipolar cells at ONL. (e) Representative immunofluorescent staining showed the activation of Müller glial (GFAP) and microglial (Iba-1) cells in old-aged retina. (f) Senescence-associated-*β*-galactosidase (SA-*β*-gal) staining of retinal ganglion cell layer (GCL) (arrows) of the 24-month-old mouse retina. The staining was absent in the retina of young mice. Scale bar: 100 *μ*m.

The effect of mTORC1 activation on aging has been demonstrated in many cells and tissues [21]. To test whether retina aging is also associated with changes of mTORC1 activity, we compared the expression of total and phosphorylated ribosomal protein S6 (S6 and p-S6, respectively) and S6 kinase (S6K and p-S6K, respectively) in 3- and 24-month-old C57BL/6 mouse retinal tissue. Western blot analysis showed that the 24-month-old mouse retina expressed higher levels of p-S6 and p-S6K than the 3-month-old ones, while the total S6 and S6K proteins remained the same between the two groups (Figures 1(a) and 1(b)). Immunofluorescent staining of p-S6 protein revealed strong positive signals in GCL and sporadic positive signals at the INL of the 24-month-old retina, while weak and sporadic staining was found in the GCL of the 3-month-old retina (Figure 1(c)). Costaining of p-S6 with markers for cells of the inner retina showed that ganglion cells and bipolar and amacrine cells expressed p-S6 in the old-aged retina (arrowheads in Figure 1(d)). These results indicated the activation of mTORC1 in the inner retina of old-aged mice, which was consistent with previous report [12]. We also found that the bipolar cells of the 24-month-old retina had aberrant dendritic tips ending at outer nuclear layer (ONL) (arrows in Figure 1(d)), which was also consistent with previous report [31]. In addition, GFAP and Iba-1 staining showed the activation of Müller glial and microglial cells in old-aged retina, respectively (Figure 1(e)).

Next, we performed SA-*β*-Gal staining on 3- and 24-month-old retina. We found positive staining only in ganglion cells of the old-aged retina (Figure 1(f)). Taken together, the results showed that old-aged retina was featured with increased mTORC1 signaling in the inner retina, activation of microglia and Müller glial cells, abnormal bipolar cell morphology, and ganglion cell senescence.

### 3.2. Generation and Characterization of *Tsc1*-cKO Mice

**Figure 2:**
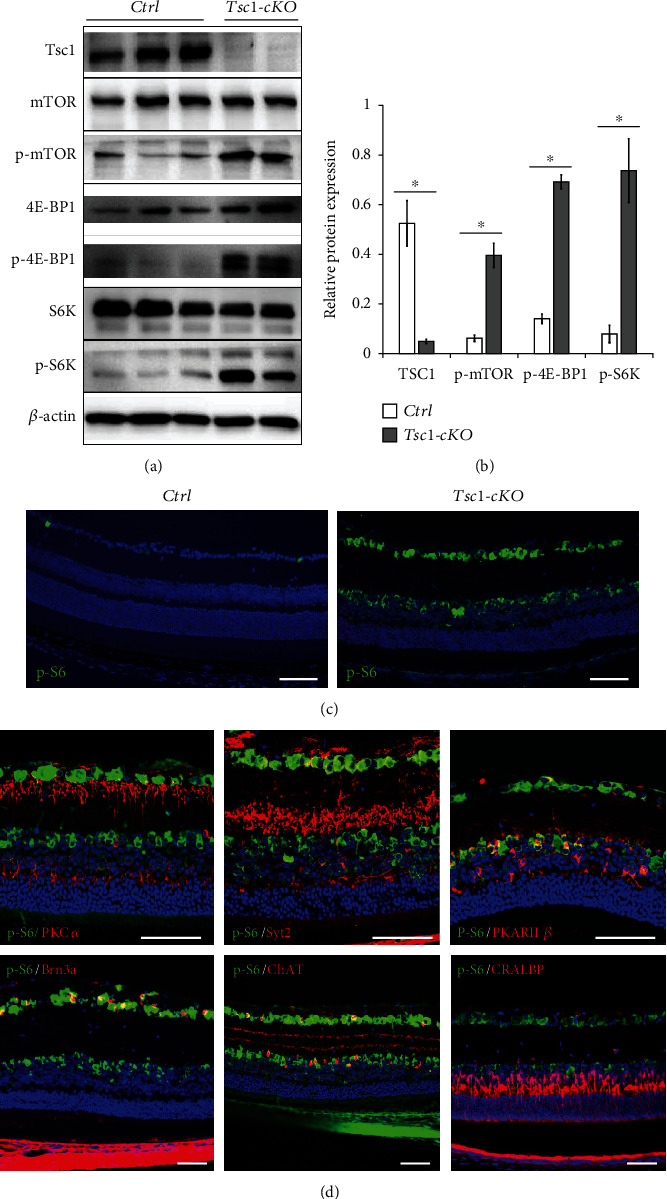
Characterization of Tsc1-cKO retina. (a) Western blot analysis of Tsc1, mTOR, phospho-mTOR (p-mTOR), 4E-BP1, phospho-4E-BP1 (p-4E-BP1), S6K, and p-S6K in total protein lysates of the Tsc1-cKO and control retina. The experiment was repeated twice. In total, 3 controls and 3 Tsc1-cKO retina samples were analyzed. (b) Relative quantification of protein expression. The density of the bands was normalized against actin for loading control and corresponding total protein for phosphorylated proteins. ^∗^*p* < 0.05 by unpaired Students' *t*-test between control and Tsc1-cKO. (c) Staining of p-S6 indicated the activation of mTORC1 in Tsc1-cKO retina was predominantly at GCL and inner part of the INL layers. Only sporadic staining was found at GCL of control retina. (d) Immunofluorescent staining revealed the localization of p-S6 in bipolar cells (PKC*α*, Syt2, PKARII*β*), ganglion cells (Brn3a), and some of the amacrine cells (ChAT) of the 3-4-month-old Tsc1-cKO retina. Weak staining was found in Müller glial cells (CRALBP). Scale bar: 100 *μ*m.

In order to understand the impact of mTORC1 activation on retina aging, we generated the *Tsc1*-cKO mice. Real-time PCR analysis of retina RNA showed that *Tsc1* gene expression in *Tsc1*-cKO retina was about 26% of controls (data not shown). Western blot analysis revealed 90% reduction of Tsc1 protein in the *Tsc1*-cKO mouse retina compared to littermate controls (Figures 2(a) and 2(b)). At the meantime, increased expression of phosphorylated mTOR, 4E-BP1, and S6 kinase was found in *Tsc1*-cKO retina, indicating the activation of mTORC1 pathway (Figures 2(a) and 2(b)).

*Chx10* is expressed in retinal progenitor cells during retina genesis and is gradually restricted to bipolar cells in adult mice [8]. *Chx10*-cre was known to be expressed in bipolar and a significant number of Müller glial cells [9, 32]. To determine cells with *Chx10*-cre expression in this study, we used *Rosa26*-tdTomato reporter mice. Red fluorescent signal was found in mosaic pattern in embryonic and neonatal retina of *Chx10*-cre; *Rosa26*-tdTomato mice (Supplementary Figure S1). In postnatal day 10 (P10) mouse retina, tdTomato expression was found in bipolar cells, Müller glial cells, some of the amacrine, horizontal, and RGC cells (Supplementary Figure S1A-G). In 4-week-old *Chx10*-Cre mouse, red fluorescent signal was concentrated in the inner retina, and the expression of Cre was restricted to cells at the outer part of the INL, presumably bipolar cells (Supplementary Figure S2H-I). These results were consistent with previous reports [9, 33, 34], suggesting that *Chx10*-cre likely led to *Tsc1* ablation in bipolar cells, a significant number of Müller glial cells, some of the RGC, amacrine, and horizontal cells in *Tsc1*-cKO retina.

To determine if *Tsc1* ablation was associated with neural retinal cell mTORC1 activation, we performed p-S6 immunofluorescent staining in 2-month-old *Tsc1*-cKO retina and found that strong positive signals were predominantly localized at GCL and the inner part of the INL (Figure 2(c)). Costaining revealed p-S6 expression in ganglion cells, bipolar cells, and a few of the amacrine cells (Figure 2(d)). We noticed very weak p-S6 staining in Müller glial cells. This was consistent with the results from a recent study showing that Müller glial cells predominantly express mTORC2 [12]. No significant p-S6 staining was found in photoreceptor cells. In rod- and cone-specific *Tsc1*-cKO mice, strong p-S6 staining was found in photoreceptor cells [18, 35]; therefore, the lack of p-S6 staining suggested negligible mTORC1 activation in photoreceptor cells of the *Tsc1*-cKO retina, which was consistent with the absence of *Chx10* expression in these cells. Collectively, the results showed that despite the broad expression of *Chx10*-cre during retinal development, the activation of mTORC1 was largely limited to cells of the inner retina, in particular, to bipolar cells, ganglion cells, and some of the amacrine cells.

### 3.3. Histological Changes of *Tsc1*-cKO Retina

**Figure 3:**
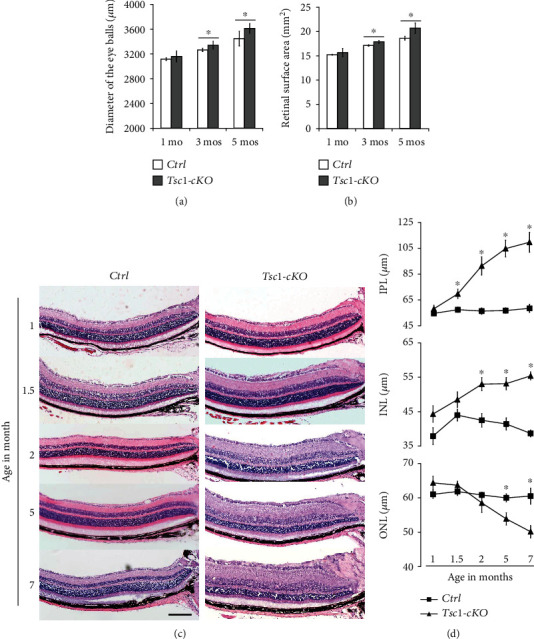
Histological changes of Tsc1-cKO retina. (a) The diameters of eye balls of 1-, 3-, and 5-month-old Tsc1-cKO and age-matched control mice. (b) Retinal surface area of 1-, 3-, and 5-month-old Tsc1-cKO and control mice. (c) H&E staining of 1-, 1.5-, 2-, 5-, and 7-month-old Tsc1-cKO and control mouse retina showing progressive thickening of the INL and IPL starting at 1.5 months and the thinning of the ONL starting at 3 months of age. (d) The average thickness of IPL, INL, and ONL of Tsc1-cKO and control retina. The thickness was measured on micrographs of H&E staining at about 500 *μ*m away from the center of the optic nerve head. At least 5 eye balls from 5 different animals in each age group of Tsc1-cKO and control were measured, and the results were averaged. Error bars represented SE. ^∗^*p* < 0.05 by unpaired Students' *t*-test between Tsc1-cKO and control group. Scale bar: 200 *μ*m.

Next we examined histological changes of the *Tsc1*-cKO retina. At the age of 1 month, no significant difference was found between *Tsc1*-cKO and control. At the age of 3 months, the eyes of the *Tsc1*-cKO were significantly bigger than the age-matched controls as indicated by the diameters of the eye balls and the surface areas of the retina (Figures 3(a) and 3(b), Supplementary Figure S2A). The surface areas of the 3- and 5-month-old *Tsc1*-cKO retina were about 4.4% and 11% bigger than controls at the same age, respectively. H&E staining of the retina at different ages showed progressive thickening of the IPL and INL layers of the *Tsc1*-cKO retina (Figures 3(c) and 3(d)). By the age of 7 months, the INL and IPL of the *Tsc1*-cKO retina were 57% and 81% thicker than the controls, respectively (Figure 3(d)). However, no retina hamartoma was found in any of the eyes examined. In contrast, the ONL layer of the *Tsc1*-cKO retina progressively thinned with age. At 5 months old, the ONL of the *Tsc1*-cKO mouse retina was 88% of the control (*p* = 0.034). The increases in the thickness of INL and ONL in the Tsc1-cKO mice were also confirmed by optical coherence tomography of animals at the ages of 3 and 5 months old (data not shown).

**Figure 4:**
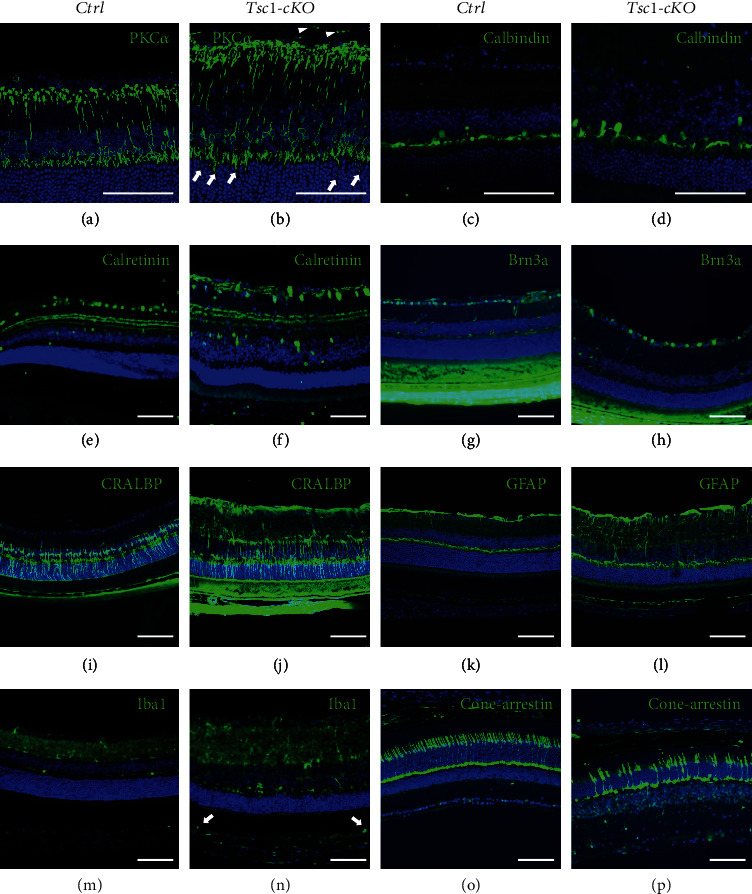
Morphological changes of the Tsc1-cKO retina. (a–l) Immunofluorescent staining of retina from 3-month-old Tsc1-cKO and control mice using antibodies against PKC*α* for rod bipolar cells (a, b), calbindin for horizontal cells (c, d), calretinin for amacrine cells (e, f), Brn3a for retinal ganglion cells (g, h), CRALBP (i, j), and GFAP (k, l) for Müller glia cells. White arrowheads and arrows in (a) indicated aberrant axon endings and dendritic tips in bipolar cells, respectively. (m–p) Immunofluorescent staining of Iba-1 and cone-arrestin of 7-month-old Tsc1-cKO and control retina to show activated microglia cells (m, n) and dramatically reduced cone photoreceptor cells (o, p). White arrows in (m) indicated Iba1-positive microglia cells in subretinal space. Scale bar: 100 *μ*m.

We further compared the numbers of nuclear at the INL between *Tsc1*-cKO and control mice at different ages. No significant differences in the density of cell nuclear was found (data not shown). However, since the diameter of the *Tsc1*-cKO eye balls was slightly larger than that of controls, we could not exclude the possibility that the mature *Tsc1*-cKO retina had slightly more cells than controls. On the other hand, significant hypertrophy of the *Chx10*-cre expressing cells was observed in the *Tsc1*-cKO retina (Figure 4). This was most evident in bipolar cells. The average cell soma of the PKC-positive rod bipolar cells from 3-month-old *Tsc1*-cKO mice was about 22% larger than the controls (Figures 4(a) and 4(b) and Supplementary Figure S2D). Aberrant endings of bipolar cell axonal projections were found at the GCL (arrowheads in Figure 4(b) and Supplementary Figure S2B). The dendrites of bipolar cells were found at the ONL at different depth (arrows in Figure 4(b), Supplementary Figure S2B), a phenomenon which was also found in old-aged retina (arrows in Figure 1(d)). To test if the aberrant dendritic tips were active, we performed CtBP2 and Psd95 staining and found positive signals for both proteins at the extended dendritic tips of *Tsc1*-cKO bipolar cells (Supplementary Figures S2B), suggesting that they were likely active in electro-signal transduction. In addition, hypertrophy of horizontal cells, amacrine, and ganglion cells were also observed (Figures 4(c)–4(h)).

Next we analyzed the effect of mTORC1 activation on glial cells. CRALBP staining showed that Müller glial cells traversed the entire thickness of the *Tsc1*-cKO retina, indicating cell hypertrophy (Figures 4(i) and 4(j)). We observed positive GFAP staining in 6-week-old *Tsc1*-cKO retina (Supplementary Figure S2C). As the *Tsc1*-cKO grew older, the staining of GFAP became stronger, suggesting persistent Müller glial cell activation (Figures 4(k) and 4(l)). Iba-1 staining revealed increased number of microglial cells in the IPL of 6-week-old *Tsc1*-cKO retina (Supplementary Figure S2C). As the *Tsc1*-cKO grew older, more microglial cells with bigger cell soma, shorter and thicker processes, and less ramification were found at the IPL and INL. In 7-month-old *Tsc1*-cKO mice, activated microglial cells were found in the subretinal space, a phenomenon which was a hallmark of retina degeneration (Figures 4(m) and 4(n)).

While the inner retina of the *Tsc1*-cKO mice showed progressive thickening, the ONL became thinner with age. Cone-arrestin staining revealed progressive decrease of cone cell density with age in the *Tsc1*-cKO retina (Figures 4(o) and 4(p), and Supplementary Figures S3A and S3B). It was reported that the thinning of ONL in normal old mouse retina was the result of expanded retina surface [36]. Even though the KO mice were bigger than the control, the percentage of retinal surface increase between *Tsc1*-cKO and control mice was similar; therefore, the thinning of the ONL in the *Tsc1*-cKO retina most likely represented the loss of photoreceptor cells.

### 3.4. Expression of Senescence-Associated Proteins and SA-*β*-Gal Staining in *Tsc1*-cKO Mouse Retina

**Figure 5:**
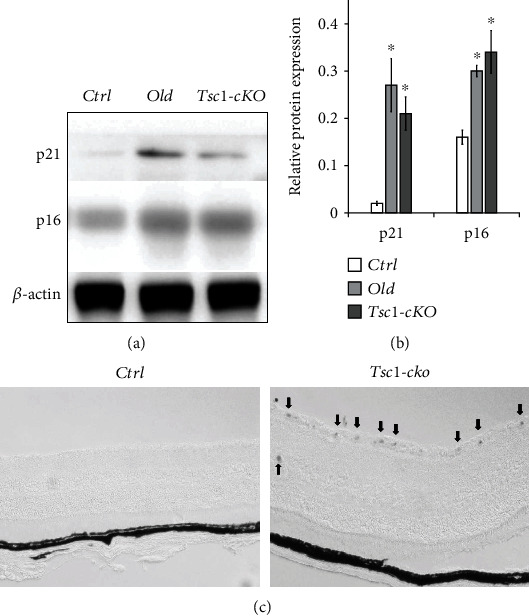
Expression of senescence-related markers in Tsc1-cKO retina. (a, b) Western blot analysis showed the upregulation of p21 and p16Ink4a protein in 5-month-old Tsc1-cKO and 24-month-old C57BL/6 (old) retina compared to age-matched control (Ctrl). The experiment was repeated twice with two different samples in each group. ^∗^*p* < 0.05 by unpaired Students' *t*-test between groups. (c) SA-*β*-gal staining of the 5-month-old Tsc1-cKO retina and age-matched control. Strong positive signals were found in GCL and INL (arrows in (c)) of the Tsc1-cKO retina. No distinctive signal was found in photoreceptor cells. Scale bar: 100 *μ*m.

To test if the activation of mTORC1 accelerated the development of age-related changes in the *Tsc1*-cKO mouse retina, we compared the expression of p16^Ink4a^ and p21 proteins among 5-month-old *Tsc1*-cKO, age-matched control, and 24-month-old C57BL/6. Western blot analysis showed that both proteins were significantly upregulated in 5-month-old *Tsc1*-cKO and 24-month-old C57BL/6 retina compared to controls (Figures 5(a) and 5(b)). SA-*β*-Gal staining revealed strong positive signals in ganglion cells of the 5-month-old *Tsc1*-cKO retina (Figure 5(c)). No distinctive signal was found in cells of the INL or photoreceptor cells. These findings were similar to what we have observed in 24-month-old C57 BL/6 mouse retina (Figure 1(f)). The results suggested that mTORC1 activation stimulated ganglion cell senescence.

### 3.5. Increased Oxidative Stress in *Tsc1*-cKO Retina

**Figure 6:**
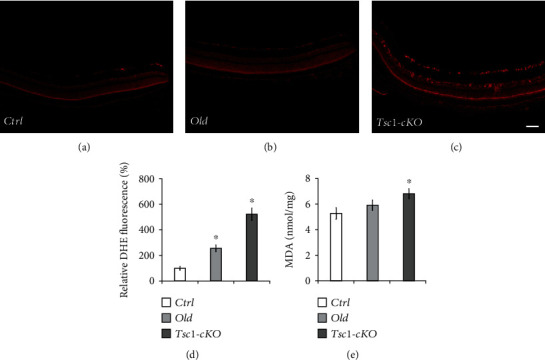
Increased oxidative stress in Tsc1-cKO retina. In vivo detection of ROS production via intraperitoneal injection of DHE revealed that retina sections from 5-month-old Tsc1-cKO mice (c) and 24-month-old C57BL/6 (old) (b) produced more ROS than age-matched control (Ctrl) mice (a). The staining was repeated on three different animals in each group. (d) The average DHE fluorescent signal intensities in Tsc1-cKO, old-aged, and control retina. The fluorescent intensity was quantified using ImageJ. The averaged signal intensity of the control group was set as 100%. Error bar represented SE. (e) Quantification of MDA in the retina of Tsc1-cKO, control, and old C57BL/6 group. Three retina samples of each group were analyzed, and the results were averaged. Error bar represented SE. Scale bar: 100 *μ*m. ^∗^*p* < 0.05 by unpaired Students' *t*-test between experimental group and controls.

An important feature of aging retina is the increase of oxidative stress, which often leads to the oxidation of protein, DNA, and lipid [37]. To analyze the level of reactive oxygen species, we performed DHE staining on 5-month-old *Tsc1*-cKO and compared that to age-matched control and 24-month-old C57BL/6 mouse retina. The *Tsc1*-cKO retina showed significantly stronger fluorescent signal than both control and old-aged retina, suggesting higher level of superoxide (Figures 6(a)–6(d)). Next, we compared the amount of MDA in retinal extracts of 5-month-old *Tsc1*-cKO, age-matched control, and 24-month-old C57BL/6 mice and found that the *Tsc1*-cKO retina had the highest level of MDA (Figure 6(e)). The difference between *Tsc1*-cKO and controls was statistically significant (*p* = 0.048, unpaired Students' *t*-test). Collectively, the data demonstrated that there was increased oxidative stress buildup in *Tsc1*-cKO retina.

### 3.6. *Tsc1*-cKO Retina Showed Gene Expression Features Which Resembles Old-Aged Retina

**Figure 7:**
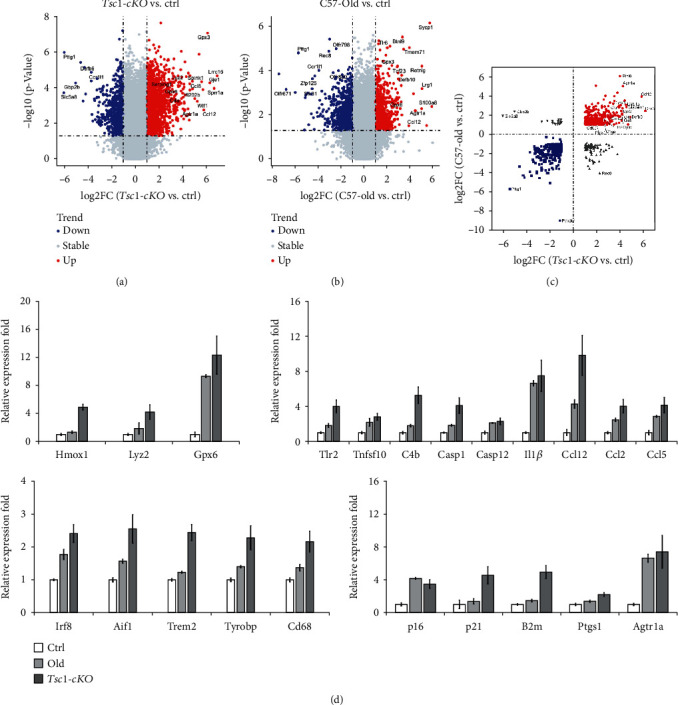
Gene expression profiles of Tsc1-cKO and 24-month-old C57BL/6 mouse retina. (a–c) Volcano plot showed the fold change of genes (log2 scale) and significance (log10 scale) between Tsc1-cKO and age-matched control mouse retina (a) and between 24-month-old C57BL/6 (old) and control (b). The vertical dotted lines indicated the threshold of fold change (log2 > 1). The horizontal dotted line indicated the threshold of the statistical significance (*p* < 0.05). The DEG for old vs. control and cKO vs. control were plotted against one another in (c). (d) Validation of microarray results with real-time PCR analysis. Fold change was relative to control and calculated as means ± SE. Each PCR was performed using two different samples and in duplicates each time, and the fold change was averaged.

To further explore the overall characteristics of the *Tsc1*-cKO retina and changes related to aging, we compared gene expression of 5-month-old *Tsc1*-cKO, age-matched controls, and 24-month-old C57BL/6 mouse retina using Agilent SurePrint G3 mouse gene expression microarray. The all-sample average detection rate was 76.9%. Using the cutoff of 2-fold change and *p* < 0.05 as criteria to select for differentially expressed genes (DEGs), we found 2210 upregulated and 1580 downregulated genes in the *Tsc1*-cKO retina compared to the age-matched controls. Between 24-month-old C57BL/6 and the controls, we found 929 upregulated and 1249 downregulated genes (Figures 7(a) and 7(b)). Some of the most up- and downregulated protein-encoding genes in two groups were denoted. The reliability of the microarray results was validated by real-time PCR analysis of selective gene expression using RNA extracted from different *Tsc1*-cKO, old-aged, and control mouse retina (Figure 7(c)). The microarray analysis showed that *Tsc1* expression in *Tsc1*-cKO retina was 28.6% of control, which was similar to the real-time PCR results. The DEG for old vs. control and cKO vs. control were plotted against one another in Figure 7(d). The number of DEGs between old-aged and control mice was more than previous report using 20- and 3-month-old C57BL/6 [38], while most of the DEGs reported in the previous study were also identified here. The difference was likely because the control we used here was *Chx10-Cre*;*Tsc1^+/+^* mice. It is also possible that more genes showed altered expression between 20- and 24-month-old retinas.

**Figure 8:**
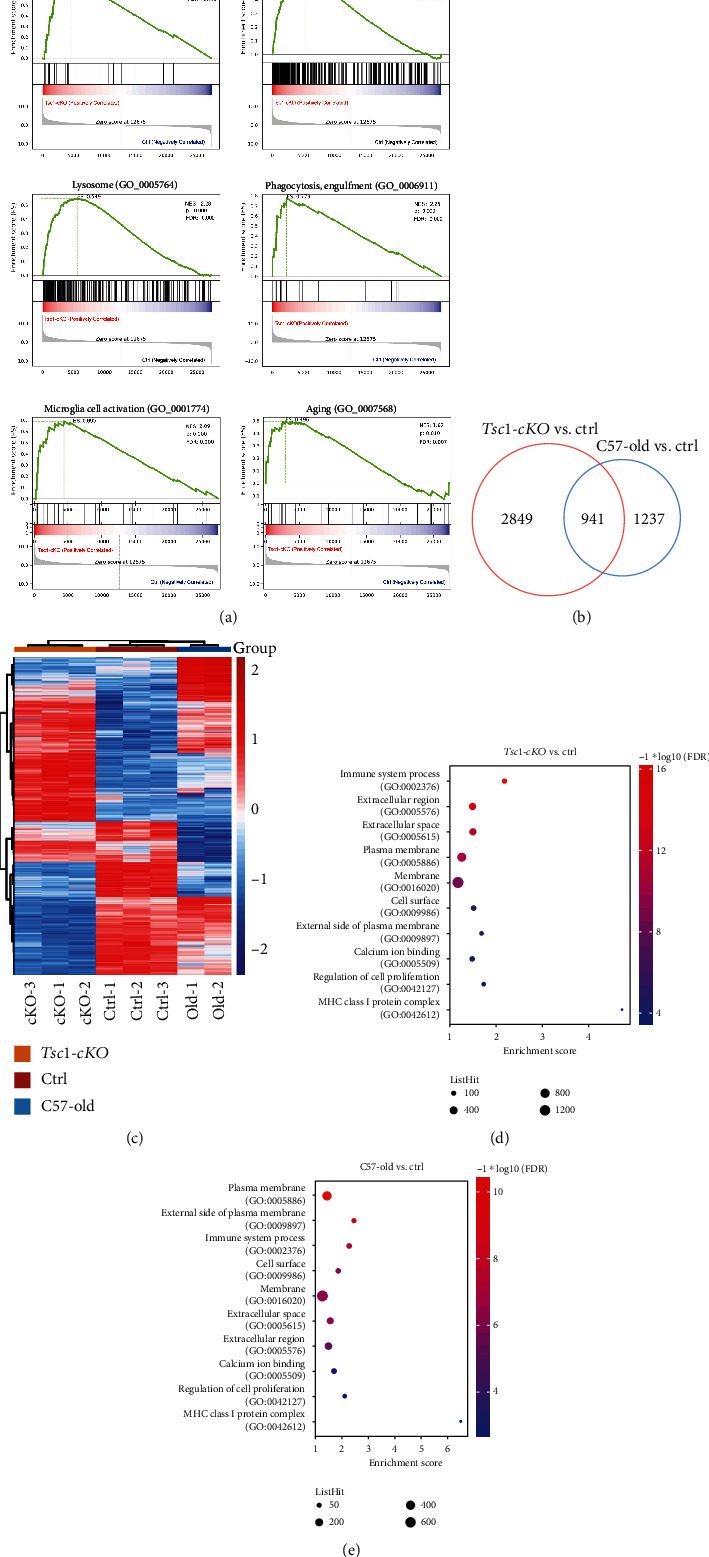
The 5-month-old Tsc1-cKO retina showed gene expression profile characteristic of old-aged retina. (a) Gene set enrichment analysis (GSEA) of DEGs between Tsc1-cKO mice and control mice. Diagrams represented selected gene sets enriched in Tsc1-cKO retina. (b) The Venn diagram of overlap in DEGs between Tsc1-cKO mice and control and old-aged C57BL/6 and control. (c) Hierarchical clustering analysis of all DEGs from Tsc1-cKO mice and control and old-aged C57BL/6 and control. (d) Gene Ontology (GO) analysis showing enrichment of the ten common GO terms in Tsc1-cKO (top) and 24-month-old (bottom).

GSEA analysis of DEGs in the *Tsc1*-cKO retina showed statistically significant enrichment of aging, microglia activity, immune system, chemokine activity, chemokine-mediated signaling pathway, and lysosome in *Tsc1*-cKO compared to control retina (Figure 8(a)). Next we identified 941 common DEGs between the *Tsc1*-cKO and old-aged groups (Figure 8(b)). The hierarchical clustering analysis of all DEGs (5027 genes in total) was shown in Figure 8(c). The top 5 most upregulated genes in *Tsc1*-cKO retina which were also significantly upregulated in the old-aged group compared to control were *Gpx3* (71-fold in *Tsc1*-cKO; 5.4-fold in old-aged), *Ccl12* (56.8-fold in *Tsc1*-cKO; 14.9-fold in old-aged), *Opticin* (40.2-fold in *Tsc1*-cKO; 3.1-fold in old-aged), *Glycam1* (23.3-fold in *Tsc1*-cKO; 7.1-fold in old-aged), and *Lilrb4* (22.6-fold in *Tsc1-*cKO; 8.4-fold in old-aged). They were involved in redox homeostasis and chemotactic responses. Among the downregulated genes, *Pttg1*, which encodes Securin, a protein which is involved in chromosome stability, was most downregulated in both groups (-93.7-fold in *Tsc1-*cKO; -48.5 fold in old-aged).

Gene Ontology (GO) analysis on the DEGs of the *Tsc1*-cKO and old-aged groups also revealed significant similarity. We used the FDR < 0.01 as cutoff threshold to select for significant GO terms and found 49 and 12 terms enriched in *Tsc1*-cKO and old-aged groups, respectively (Supplementary Table S3). Among these terms, 10 were in common. The enrichment scores of the 10 common terms in each group were plotted in Figures 8(d) and 8(e). The GO terms and representative genes included immune system process (GO:0002376; *Ccl12*, *Agtr1a*, *Cfb*, *C3*, *Cd163*, etc.), extracellular region (GO:0005576; *Gpx3*, *Optc*, *Defb9*, *Glycam1*, *Ltbp2*, etc.), and calcium ion binding (GO:0005509; *Tnnt2*, *Adgre1*, *Mctp1*, *Tgm2*, *S100a6*, etc.). A number of the GO terms which were found significantly involved only in *Tsc1-*cKO mice were also related to inflammation/immune responses (GO:0006954; *Tlr1*, *Oasl1*, *Ccl4*, *Nlrc5*, *Cd55*, etc.), chemotaxis (GO:0006935; *Pf4*, *Ccl5*, *Cxcl13*, *Ccl4*, *Ccl2*, etc.), and extracellular matrix (GO:0031012; *Gpx6*, *Wif1*, *Scrg1*, *Colec10*, *Crisp1*, etc.). In addition, there were alterations in the regulation of cell apoptosis (GO:0042981; *Tnfsf10*, *Casp1*, *Bcl2a1c*, *Bid*, *Casp12*, etc.), oxidoreductase activity (GO:0016491; *Frrs1*, *Ptgs1*, *Ncf1*, *Hmox1*, *Pyroxd2*, etc.), and aging (GO:007568; *Agtr1a*, *Pttg1*, *B2m*, *Apoe*, *Apod*, etc.) in *Tsc1-*cKO mice. Collectively, the data suggested that the activation of mTORC1 signaling caused disruption in retinal homeostasis.

### 3.7. Progressive Reduction of ERG Responses with Age in *Tsc1*-cKO Mice

**Figure 9:**
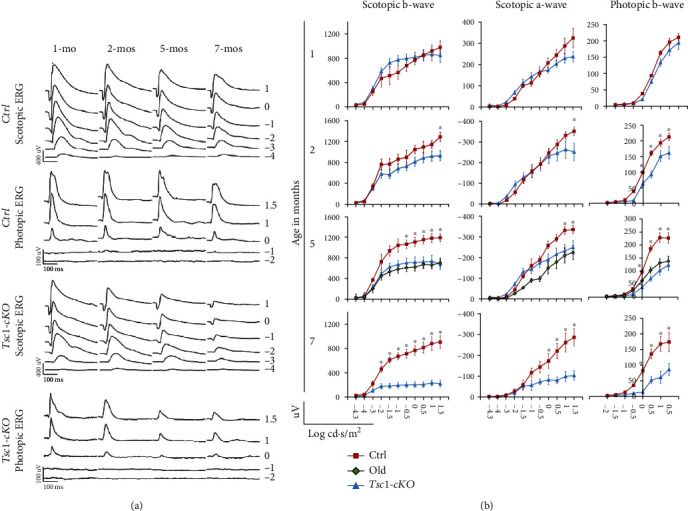
ERG responses of Tsc1-cKO, age-matched control, and 24-month-old C57BL/6 mice. (a) Representative full-field scotopic and photopic ERG responses of the Tsc1-cKO and control mice at 1, 2, 5, and 7 months of age. Rapid decrease of both scotopic and photopic responses was observed in the Tsc1-cKO mice. (b) Average a- and b-wave amplitudes at different units of luminance in Tsc1-cKO and control mice by age. The age of each group was indicated at the left side. The responses of 24-month-old C57BL/6 (old) were compared to that of the 5-month-old Tsc1-cKO and control. At least 6 mice in each age group of each genotype were tested. The error bars represented the SE. ^∗^*p* < 0.05 by unpaired Students' *t*-test between Tsc1-cKO and control.

Retina aging contributes to the deterioration of visual function [1]. To evaluate the function of *Tsc1*-cKO mouse retina, we conducted full-field scotopic and photopic ERG on *Tsc1*-cKO and control mice (Figures 9(a) and 9(b)). At 1 month, there was no significant difference in scotopic or photopic ERG responses between *Tsc1*-cKO and controls. At 2 months, the *Tsc1*-cKO exhibited smaller b-wave amplitude than the littermate controls at 1 and 1.3 log cd∙s/m^2^ under scotopic conditions, suggesting abnormal bipolar cell activity. The reduction in b-wave amplitude progressed as the *Tsc1*-cKO became older. At the age of 5 months, differences between *Tsc1*-cKO and control were seen at lower flash unit of luminance. In addition, the decrease of a-wave amplitude in *Tsc1*-cKO became significant. To better isolate the activities of photoreceptor cells in the ERG [39], we also compared the amplitudes at 8 ms after the flash to exclude the potential contribution from the inner retina cells and found that the *Tsc1*-cKO had significantly reduced amplitudes than controls (Supplementary Figure S3C). At the age of 7 months, the maximal a- and b-wave amplitudes of *Tsc1*-cKO were about 36% (*p* = 0.034) and 24.3% (*p* = 0.009) of those of the control mice, respectively. A similar decrease in the amplitudes at 8 ms after the flash was also observed in the *Tsc1*-cKO mice.

Consistent with the reduced scotopic responses, decreased b-wave amplitude was also observed in *Tsc1*-cKO under photopic conditions. As comparison, we also measured ERG responses in 24-month-old C57BL/6 mice. The results showed that the ERG responses of the 5-month-old *Tsc1*-cKO were comparable to those of the 24-month-old C57BL/6 mice (Figure 9(b)).

### 3.8. Rapamycin Treatment Largely Reverted the Abnormalities Observed in *Tsc1-*cKO Retina

**Figure 10:**
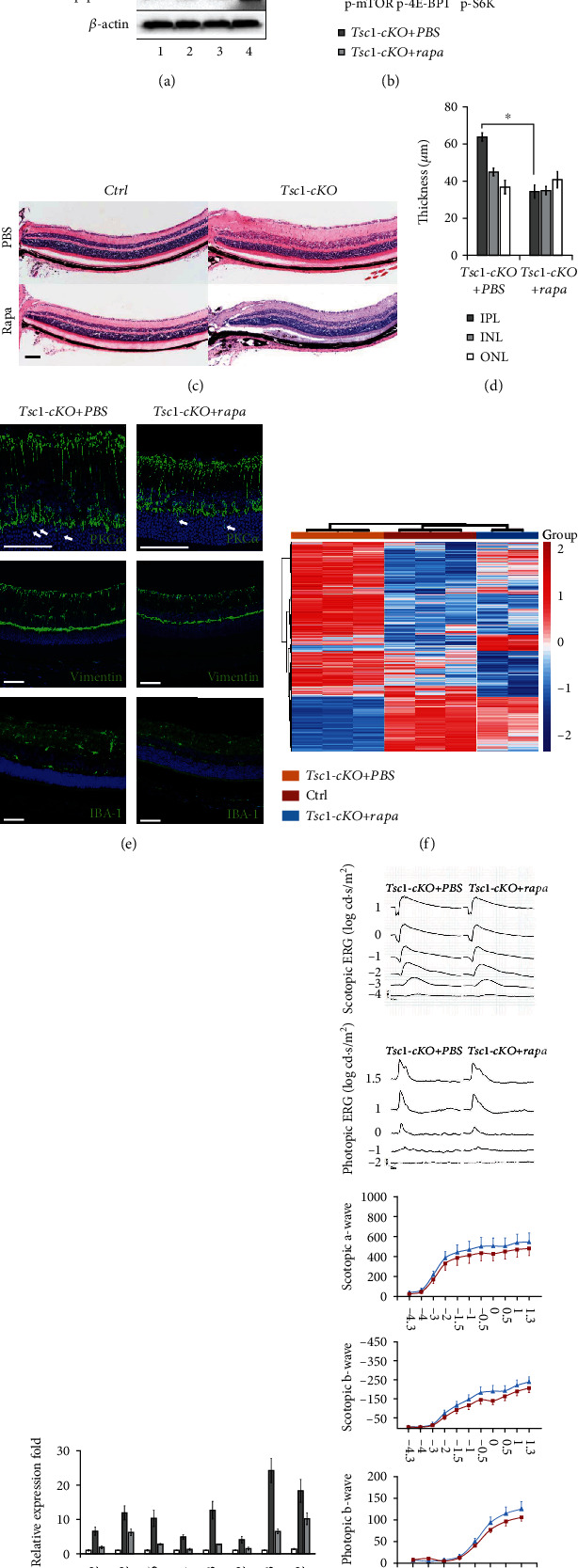
The effect of rapamycin on Tsc1-cKO retina. (a) Representative Western blot analysis of total and phosphorylated form of mTOR, 4E-BP1, and p70S6K in total retinal extracts of Tsc1-cKO and control mice after rapamycin (Rapa) or PBS treatment for 1 month. Rapamycin treatment successfully downregulated mTORC1 signaling in the Tsc1-cKO retina. (b) Relative quantification of protein expression. The density of the bands was normalized as described above. ^∗^*p* < 0.05 by unpaired Students' *t*-test between Tsc1-cKO and control group. The experiments were repeated three times with one pair of samples at each time. (c) Representative H&E staining of 3-month-old Tsc1-cKO and control mouse retina after rapamycin- or PBS-treatment. (d) The average thickness of INL, IPL, and ONL of Tsc1-cKO mice after rapamycin or PBS treatment. The thickness was measured as described in Figure 3. Data represented the average reading from 4 animals in each group. Error bars represented SE. ^∗^*p* < 0.05 by unpaired Students' *t*-test between PBS and rapamycin-treated group. Scale bar: 200 *μ*m. (e) Immunofluorescent staining of PKC*α* (top panel), Vimentin (middle panel), and Iba-1 (bottom panel) on Tsc1-cKO retina after rapamycin (right column) or PBS (left column) treatment. (f) Hierarchical clustering analysis of gene expression profiles of control, Tsc1-cKO, and rapamycin-treated Tsc1-cKO retina. (g) Expression of representative genes in rapamycin-treated and untreated Tsc1-cKO retina. Fold change was relative to control and calculated as means ± SE. (h) Representative full field ERG responses of Tsc1-cKO mice after PBS or rapamycin treatment. The a-wave and b-wave amplitudes in response to increasing flash intensity were presented at the lower part of the panel. The data represented the averaged readings from 4 animals in each group. Error bars represented SE. Unpaired Students' *t*-test was performed to compare the differences between PBS- and rapamycin-treated animals at each light intensity; no significant differences were found.

To verify that the abnormalities observed in *Tsc1*-cKO retina were indeed due to mTORC1 activation, we used rapamycin to inhibit mTORC1 signaling in *Tsc1*-cKO mice. Intraperitoneal injection of rapamycin effectively reduced the phosphorylation of mTOR, 4E-BP1, and S6K in the retina of *Tsc1*-cKO mice (Figures 10(a) and 10(b)). When we treated young *Tsc1*-cKO mice with rapamycin before they developed significant morphological abnormalities, we found that rapamycin effectively prevented the thickening of INL and IPL (data not shown). We then treated 3-month-old *Tsc1*-cKO mice (which already showed significantly thicker INL and IPL than the age-matched controls) with rapamycin and found that it effectively reversed the thickened INL and IPL. At the end of the treatment, the INL and IPL layers were significantly thinner than the PBS-treated *Tsc1*-cKO mice (Figures 10(c) and 10(d)). On the other hand, rapamycin treatment had no significant effect on the retinal thickness of control animal (Figure 10(c)). PKC*α* and Vimentin staining revealed shortened bipolar and Müller glia in *Tsc1*-cKO retina after rapamycin treatment (Figure 10(e)), suggesting that the thinning of the INL and IPL was most likely due to the shortening of the secondary neurons. However, improper dendritic tip arborization of bipolar cells at ONL remained (Figure 10(e), white arrows).

The rapamycin-treated *Tsc1*-cKO mice also showed less Iba-1 positive cells than the PBS-treated retina (Figure 10(e)), suggesting reduced microglial cell activation in *Tsc1*-cKO retina after rapamycin treatment. We further compared the RNA expression profile of the rapamycin-treated *Tsc1*-cKO retina to that of control and 5-month-old *Tsc1*-cKO retina without treatment. We used 4-month-old *Tsc1*-cKO for rapamycin treatment because the retina already showed signs of old-aged animal. The results showed that the RNA expression profile of the rapamycin-treated retina was similar to that of controls and very different from that of the *Tsc1*-cKO (Figure 10(f)). Furthermore, the expression of inflammation-related genes was significantly reduced in rapamycin-treated *Tsc1*-cKO retina compared to *Tsc1*-cKO without treatment (Figure 10(g)).

However, the ERG responses of the rapamycin-treated 4-month-old *Tsc1*-cKO were slightly reduced than that of the PBS-treated *Tsc1*-cKO mice, but the differences were not statistically significant (Figure 10(h)). Interestingly, rapamycin also caused small reduction of ERG responses in control mice. We speculated that the lack of ERG improvement was associated with the remaining of aberrant dendritic tip endings at the ONL of the rapamycin-treated retina. In another study using the knockout of Pten to activate mTORC1 signaling, the authors found that rapamycin treatment could not reverse the aberrant migration of granule cells at dentate gyrus [40]. It is also possible that the toxic effect of rapamycin as previously reported in rabbit eye was responsible for the lack of rescuing effect here [41].

### 3.9. Inhibition of Microglial Cell Activity by Minocycline Prevented Photoreceptor Cell Loss and Partially Restored ERG Function in the *Tsc1*-cKO Mice

**Figure 11:**
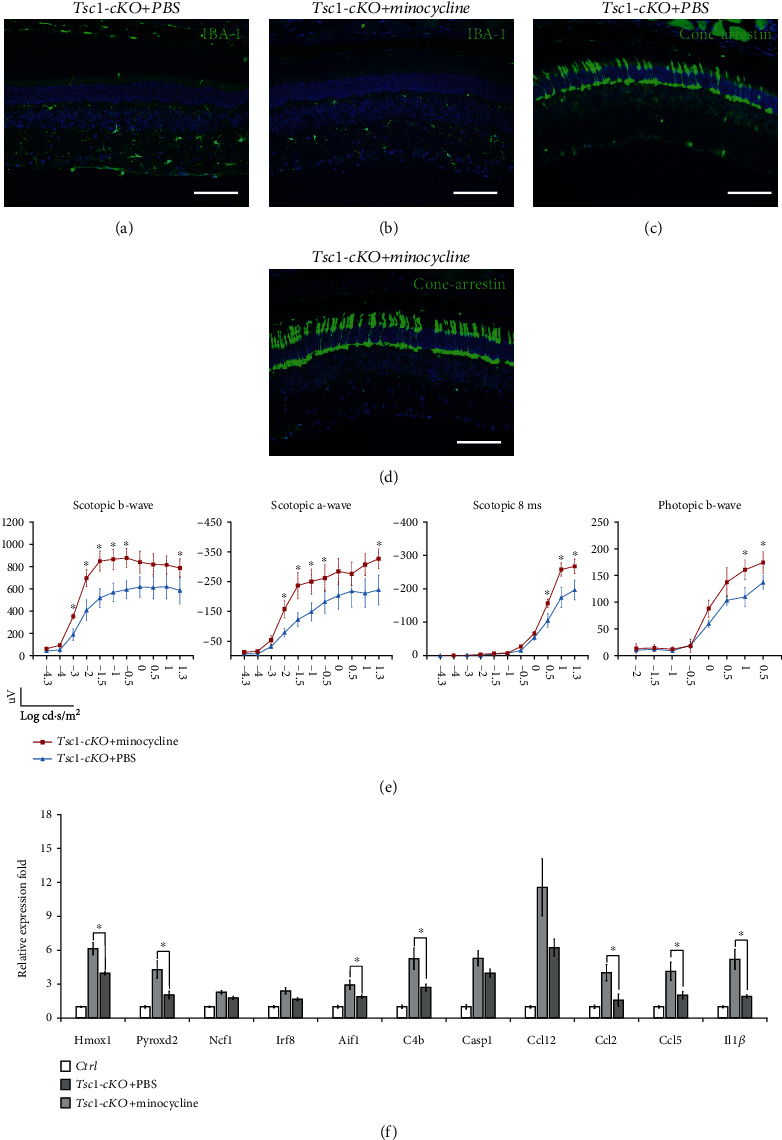
The effect of minocycline on Tsc1-cKO mouse retina. (a, b) Retina sections of Tsc1-cKO retina with or without minocycline treatment were stained with Iba-1 to show the inhibition of microglial cells after minocycline treatment. (c, d) Cone-arrestin staining of the Tsc1-cKO retina with and without minocycline treatment. The minocycline-treated retina showed more arrestin-positive cells, suggesting that the inhibition of microglial cells prevented photoreceptor cell loss. The staining was performed on 6 retina from 6 different animals, 3 in each group. (e) ERG responses of Tsc1-cKO mice with or without minocycline treatment. Three animals in each group were subjected to ERG analysis, and the a-wave, b-wave, and responses at 8 ms after the flash were averaged. Error bars represented SE. ^∗^ marked significant differences of *p* < 0.05 by unpaired Students' *t*-test at the indicated luminance. (f) Real-time PCR analysis of genes associated with oxidative stress (Hmox1, Pyroxd2, Ncf1), microglia activity (Irf8, Aif1, C4b), chemotaxis activity (Ccl12, Ccl2, Ccl5), and inflammatory response (Casp1, Il1*β*) in Tsc1-cKO retina with or without minocycline treatment. The fold change was relative to untreated- and age-matched control. Six total RNA samples, 3 in each group, were used for PCR analysis. ^∗^*p* < 0.05 by unpaired Students' *t*-test between minocycline- and PBS-treated groups.

Activated microglial cell is believed to stimulate retina aging [42]. Compared to old-aged retina, the *Tsc1*-cKO retina showed a higher degree of microglial cell activation as indicated by Iba-1 staining (Figures 1(e) and 4(m)). To determine the role of activated microglial cells in the development of the abnormalities observed in *Tsc1*-cKO retina, we treated 4-months-old *Tsc1*-cKO mice with minocycline for 4 weeks. The minocycline-treated *Tsc1*-cKO retina showed less microglial cell accumulation at the IPL and OPL than the PBS-treated *Tsc1*-cKO retina, and the cells were also more ramified (Figures 11(a) and 11(b)), indicating successful inhibition of microglial cell activation. Furthermore, cone-arrestin staining showed that the minocycline-treated *Tsc1*-cKO retina had higher cone cell density than the PBS-treated ones, suggesting that minocycline treatment prevented the loss of photoreceptor cells in the *Tsc1*-cKO retina (Figures 11(c) and 11(d)). ERG analysis showed that the minocycline-treated *Tsc1*-cKO had significantly bigger a-wave and b-wave amplitudes than the PBS-treated *Tsc1*-cKO under scotopic conditions (Figure 11(e)). Measurement of responses at 8 ms after the flash also revealed a bigger amplitude in minocycline-treated *Tsc1*-cKO mice.

Next we compared the expression of selective oxidative stress- and inflammation-related genes in minocycline and PBS-treated *Tsc1*-cKO retina to control retina and found that the oxidative stress-associated genes such as *Homx1* and *Pyroxd2* and inflammation-related genes such as *Aif1*, *C4b*, *Ccl2*, *Ccl5*, and *Il1β* were significantly downregulated in the minocycline-treated group (Figure 11(f)). Collectively, these results suggested that the activation of microglial cells was at least partially responsible for the functional loss of *Tsc1*-cKO retina.

## 4. Discussion

This study investigated the role of mTORC1 signaling in retina aging in old-aged C57BL/6 and *Tsc1*-cKO mice. We showed that mTORC1 signaling was upregulated in naturally aged C57BL/6 retina, and the upregulation of mTORC1 signaling in *Chx10*-expression retinal cells accelerated the aging process and caused retinal degeneration. This was evidenced by selective cell senescence, accumulation of oxidative stress, activation of microglial cells, a gene expression profile which bears signatory features of aging retina, and profound decline in retinal function.

We found that mTORC1 activation was most noticeable in ganglion cells of naturally aged retina and mTORC1 activation accelerated ganglion cell aging. The results were consistent with previous study which showed prominent expression of typical senescence-related proteins in ganglion cells, less in cells of the INL, such as amacrine, and Müller glial cells and limited in rod photoreceptor cells [4]. Single-cell RNA sequencing of primate retina also showed that RGC cells had most DEGs between old-aged and young animals [43]. Other studies have shown that the activation of mTORC1 was required for axonal regrowth after ganglion cell trauma [9, 13]. In fact, the optic nerve fiber of the Chx10-cre mice was also thicker than that of the controls (data not shown). Collectively, these results demonstrated an important role of mTORC1 signaling in ganglion cell homeostasis and aging.

Our study also suggested that mTORC1 activation is involved in age-related changes of bipolar cells. In 24-month-old C57BL/6 mouse retina, we found that the dendritic tips of the PKC*α*-positive rod bipolar cells extended to the ONL, a phenomenon which was also reported in an earlier study [31]. This phenomenon was replicated in *Tsc1*-cKO retina with significant cell hypertrophy. Abnormal extention of the bipolar cell dendritic tips to the ONL was found when there were photoreceptor cell contraction or defective synpatic protein synthesis [44–47]. In these circumstances, the extension of bipolar cell processes was likely an adaptive response to restore visual signal transduction. However, this is not likely the case in *Tsc1*-cKO retina, since photoreceptor cells were not the primary affected cells and the thinning of ONL layer occurred at a much later stage. Therefore, it is tentative to propose that activation of mTORC1 stimulated the growth of bipolar cell processes which resulted in abnormal positioning of the dendritic endings and contributed to defective visual-signal transduction.

Despite the expression of *Chx10* in significant number of Müller glial cells, we observed very faint p-S6 staining in Müller glial cells of the *Tsc1*-cKO retina. The result suggested limited mTORC1 activation and was consistent with a previous report which showed that mouse Müller glia predominantly express mTORC2 [12]. However, Müller glial cell hypertrophy and activation was evident in *Tsc1*-cKO retina starting at 6 weeks of age, at the same time when bipolar cells start showing hypertrophy. At the present time, we were not able to tell if Müller gliosis were the direct consequence of altered mTORC1 signaling or the response to the hypertrophy of bipolar and other intermediate neurons. In zebrafish and chicken eyes, mTOR activation promoted the dedifferentiation of Müller glial cells and the formation of Müller glia-derived progenitor cells and subsequent retina regeneration [48, 49]. In human retina of old age, heterogeneous groups of glial cells were found by single cell transcriptomic analysis [50]. However, no significant changes of Müller glia function in old-aged retina were reported. Therefore, the role of mTORC1 signaling in Müller glia aging remains to be explored.

One of the most interesting findings of this study was the contribution of microglial cells in retinal aging and degeneration. Microglial cells play important roles in developmental, physiological, and pathophysiological processes of retina [51]. A previous study showed that the activation of microglial cells and the severity of inflammation attributed to the susceptibility of retinal degeneration in different mouse strains [52]. It was speculated that the activated microglial cells participated in the aging of retina [42, 53]. Microglial cell activation in *Tsc1*-cKO retina followed bipolar and Müller glial cell hypertrophy. In response to *Tsc1* ablation-induced mTORC1 activation, the microglia cells of the *Tsc1*-cKO retina showed more profound activation than the naturally aged retina, which correlated with more intense oxidative stress buildup and upregulation of a broad range of redox- and inflammation-related genes. Inhibition of microglial cells by minocycline in the *Tsc1*-cKO mice prevented the decrease of ERG responses and photoreceptor cell loss. The role of microglia cell in accelerating the retinal aging and degeneration represented a noncell autonomous effect of mTORC1 activation in neuronal retinal cells.

Finally, our study complemented a previous study which also used *Tsc1*-cKO mice and showed that mTORC1 activation accelerated retinal development by shorten the cell cycle time of the affected retinal progenitor cells [10]. Our results suggested that in adult *Tsc1*-cKO mice, mTORC1 activation accelerated senescence of the postmitotic, fully differentiated neuroretinal cells and ultimately led to retinal degeneration.

## 5. Conclusions

In summary, our study showed that mTORC1 activation participated in retinal aging via both cell autonomous (as in retinal ganglion cells) and nonautonomous mechanisms (largely by activated microglia cells). Our study further highlighted the role of microglia cells in driving the functional decline of the retina. Finally, we showed that the *Tsc1*-cKO mice represented an animal model of retinal degeneration and aging.

## Data Availability

The datasets supporting the conclusions of this article are available in the GEO repository (GSE177039: https://www.ncbi.nlm.nih.gov/geo/query/acc.cgi?acc=GSE177039). The datasets used and/or analyzed during the current study are available from the corresponding author on reasonable request.

## Supplementary Materials

Supplementary Materials Supplementary Table S1: antibodies used for immunofluorescent staining and Western blot. Supplementary Table S2: sequences of primers used in the study. Supplementary Table S3: GO terms significantly upregulated in 5-month-old *Tsc1*-cKO retina compared to age-matched controls. Supplementary Figure S1: identification of *Chx10*-expressing cells in postnatal mouse retina. Supplementary Figure S2: histological and morphological changes in *Tsc1*-cKO retina. Supplementary Figure S3: progressive loss of cone photoreceptor cells and the associated decrease in ERG responses in Tsc1-cKO mice.
