# Supplementary material for: mTORC1 Activation in Chx10-Specific Tsc1 Knockout Mice Accelerates Retina Aging and Degeneration
Source: Oxid Med Cell Longev. 2021 Nov 5;2021:6715758. doi: 10.1155/2021/6715758 (PMC8589503; doi:10.1155/2021/6715758)
Supplement: Supplementary Materials — Supplementary Table S1: antibodies used for immunofluorescent staining and Western blot. Supplementary Table S2: sequences of primers used in the study. Supplementary Table S3: GO terms significantly upregulated in 5-month-old Tsc1-cKO retina compared to age-matched controls. Supplementary Figure S1: identification of Chx10-expressing cells in postnatal mouse retina. Supplementary Figure S2: histological and morphological changes in Tsc1-cKO retina. Supplementary Figure S3: progressive loss of cone photoreceptor cells and the associated decrease in ERG responses in Tsc1-cKO mice. [file 6715758.f1.docx]

**Supplementary Table S1: Antibodies used in the study**

| **Target protein** | **Antibody type** | **dilution** | **Catalog No.** | **Source** |
| --- | --- | --- | --- | --- |
| p-S6(S240/244) | Rabbit mAb | 1:100 (IF)  1:1000 (WB) | 5364S | Cell Signaling Technology |
| Cre Recombinase | Rabbit mAb | 1:50 (IF) | 15036S | Cell Signaling Technology |
| Goα | Mouse mAb | 1:100 (IF) | MAB3073 | MerckMillipore, |
| PKCα | Rabbit mAb | 1:50 (IF) | 2056S | Cell Signaling Technology |
| Syt2 | Mouse mAb | 1:100 (IF) | ab154035 | Abcam |
| PKARIIβ | Mouse mAb | 1:100 (IF) | 610625 | BD |
| Calretinin | Rabbit pAb | 1:100 (IF) | GTX103261 | GeneTex |
| Calbindin | Rabbit mAb | 1:200 (IF) | 13176T | Cell Signaling Technology |
| CRALBP | Mouse mAb | 1:100 (IF) | GTX15051 | GeneTex |
| ChAT | Goat pAb | 1:200 (IF) | AB144P | MerckMillipore, |
| GFAP | Rabbit pAb | 1:200 (IF) | Z0334 | Dako |
| Brn3a | Mouse mAb | 1:20 (IF) | sc-8429 | Santa Cruz Biotechnology |
| Cone-Arrestin | Rabbit pAb | 1:100 (IF) | AB15282 | MerckMillipore, |
| Rhodopsin | Mouse mAb | 1:50 (IF) | sc57432 | Santa Cruz Biotechnology |
| IBA-1 | Rabbit pAb | 1:200 (IF) | 019-19741 | Wako |
| CtBP2 | Mouse mAb | 1:100 (IF) | NBP3-07789 | Novus Biologicals |
| PSD95 | Rabbit pAb | 1:200 (IF) | 3450S | Cell Signaling Technology |
| S6 | Rabbit mAb | 1:1000 (WB) | 2217S | Cell Signaling Technology |
| Tsc1 | Sheep pAb | 1:500 (WB) | AF4379 | R&D Systems |
| mTOR | Rabbit mAb | 1:1000 (WB) | 2972S | Cell Signaling Technology |
| p-mTOR (S2448) | Rabbit mAb | 1:1000 (WB) | 2971S | Cell Signaling Technology |
| 4E-BP1 | Rabbit mAb | 1:1000 (WB) | 9452S | Cell Signaling Technology |
| P-4E-BP1 (T37/46) | Rabbit mAb | 1:1000 (WB) | 2855S | Cell Signaling Technology |
| S6 Kinase | Rabbit mAb | 1:1000 (WB) | 9202S | Cell Signaling Technology |
| p-S6 Kinase (T389) | Rabbit mAb | 1:1000 (WB) | 9205S | Cell Signaling Technology |
| β-actin | Mouse mAb | 1:2000 (WB) | sc-47778 | Santa Cruz Biotechnology |
| CDKN1A/p21 | Rabbit pAb | 1:1000 (WB) | A1483 | Abclonal |
| CDKN2A / p16^INK4a^ | Rabbit pAb | 1:1000 (WB) | A0262 | Abclonal |

IF: Immunofluorescence; WB: Western blot.

**Supplementary Table S2 : PCR primer list**

| **Gene** | **Forward primer** | **Reverse primer** |
| --- | --- | --- |
| *Hmox1* | AAGCCGAGAATGCTGAGTTCA | GCCGTGTAGATATGGTACAAGGA |
| *Lyz2* | ATGGAATGGCTGGCTACTATGG | ACCAGTATCGGCTATTGATCTGA |
| *Gpx6* | GCCCAGAAGTTGTGGGGTTC | TCCATACTCATAGACGGTGCC |
| *Tlr2* | GCAAACGCTGTTCTGCTCAG | AGGCGTCTCCCTCTATTGTATT |
| *C4b* | ACTTCAGCAGCTTAGTCAGGG | GTCCTTTGTTTCAGGGGACAG |
| *Casp12* | AGACAGAGTTAATGCAGTTTGCT | TTCACCCCACAGATTCCTTCC |
| *Ccl12* | ATTTCCACACTTCTATGCCTCCT | ATCCAGTATGGTCCTGAAGATCA |
| *Ccl2* | TTAAAAACCTGGATCGGAACCAA | GCATTAGCTTCAGATTTACGGGT |
| *Ccl5* | GCTGCTTTGCCTACCTCTCC | TCGAGTGACAAACACGACTGC |
| *Irf8* | CGGGGCTGATCTGGGAAAAT | CACAGCGTAACCTCGTCTTC |
| *Aif1* | ATCAACAAGCAATTCCTCGATGA | CAGCATTCGCTTCAAGGACATA |
| *Trem2* | CTGGAACCGTCACCATCACTC | CGAAACTCGATGACTCCTCGG |
| *Tyrobp* | GAGTGACACTTTCCCAAGATGC | CCTTGACCTCGGGAGACCA |
| *Cd68* | CCATCCTTCACGATGACACCT | GGCAGGGTTATGAGTGACAGTT |
| *CDKN1A(p21)* | GGTGAGGAGGAGCATGAATGG | GGACATCACCAGGATTGGACA |
| *CDKN2A(p16)* | GGAAAGCGAACTCGAGGAGAG | GGGTACGACCGAAAGAGTTC |
| *B2m* | CTGGTCTTTCTGGTGCTTGTC | GTTCAGTATGTTCGGCTTCCC |
| *Ptgs1* | GCATTGCACATCCATCCACTC | AACAGGGATTGACTGGTGAGG |
| *Agtr1a* | AACAGCTTGGTGGTGATCGTC | CATAGCGGTATAGACAGCCCA |
| *Pyroxd2* | TCTGAGAGACCCCTACTCCTT | CCTTCCGTGAGAACTGAGAGA |
| *Ncf1* | CTTAGCCAGGACACCTATCGC | CGATGGATTGTCCTTTGTGCC |
| *Casp1* | CTGTATTCACGCCCTGTTGGA | CCTCAGGATCTTGTCAGCCAT |
| *IL1β* | TTCAGGCAGGCAGTATCACTC | CCCATGAGTCACAGAGGATGG |

.

**Supplementary Figure S1**

**
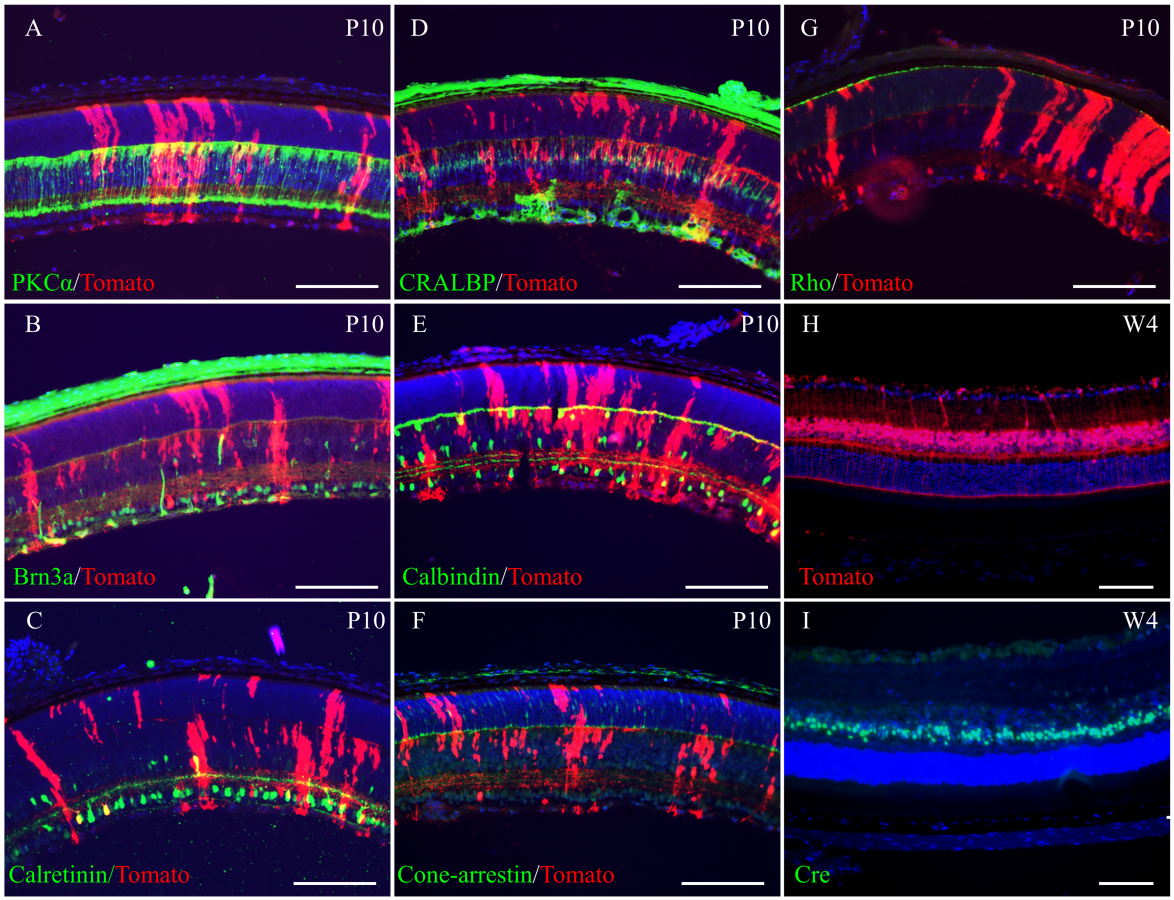
**

**Supplementary Figure S1. Identification of *Chx10*-expressing cells in postnatal mouse retina.** Immunofluorescent staining of postnatal day 10 retina from *Chx10*-cre; *Rosa^tdTomato^* mice using antibodies against PKCα for bipolar cells **(A)**, Brn3a for retinal ganglion cells (RGC)**(B)**, calretinin for amacrine cells **(C)**, CRALBP for Müller glial cells **(D)**, calbindin for horizontal cells **(E)**, cone-arrestin for cone photoreceptor cells **(F)**, and Rho for rod photoreceptor cells **(G)**. Orange signal of tdTomato was found co-localized with bipolar, some of the RGC, amacrine, Müller glial and horizontal cells, but not with photoreceptor cells. Immunofluorescent staining of tdTomato **(H)** and Cre protein **(I)** in 4-week old*Chx10*-cre; *Rosa^tdTomato^* mouse retina. The expression of Cre was exclusively localized in the INL layer. Scale bar: 100μm.

**Supplementary Figure S2**

**
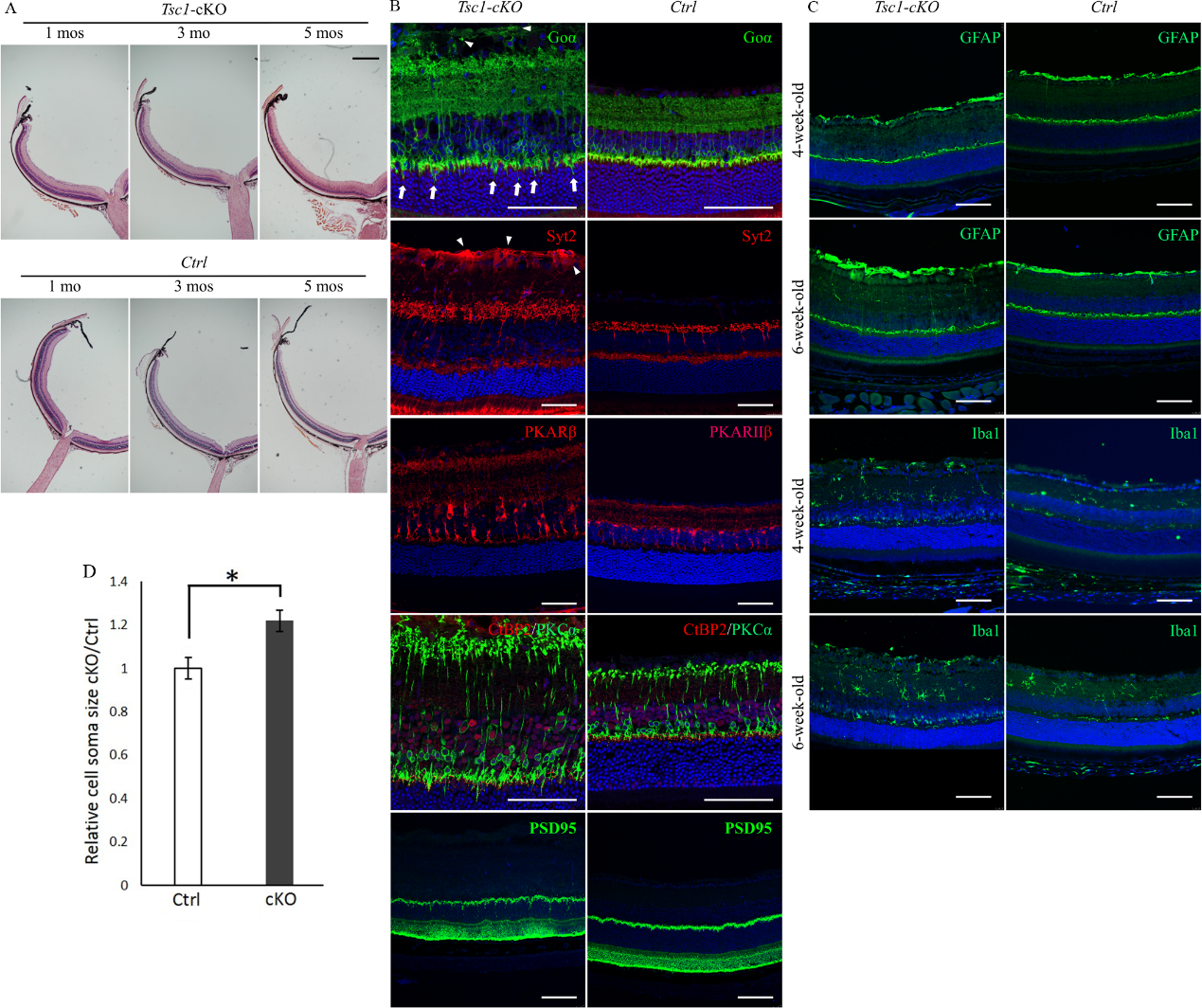
**

**Supplementary Figure S2. Histological and morphological changes in *Tsc1*-cKO retina. (A).** Representative H&E staining of the temporal retina obtained by sagittal section of 1-, 3- and 5-month old*Tsc1*-cKO and control mice retina. The retina from the knockout mice were thicker and had larger surface area than the age-matched control at 3- and 5-month of age. Scale bar: 500 μm. **(B).** Bipolar cell hypertrophy in *Tsc1*-cKO mice retina. Cryosections of the *Tsc1*-cKO and control mice retina were stained with antibodies against Goα for on-bipolar, Syt2 for off-bipolar and PKARIIβ for type III cone-bipolar cells. The cell soma of the knockout retina were larger than that of the controls. Axon endings of Goα- and Syt2-positive cells were seen at the NFL (arrowheads). Dendritic tips were observed in the ONL for Goα-positive cells (white arrows). Positive staining of CtBP2 and PSD95 were observed at the extended bipolar dendritic tips of the knockout mice, suggesting possible synaptic function. Scale bar: 100 μm. **(C).** Immunofluorescent staining of GFAP and Iba1 to show the activation of Müller glial and microglial cells in *Tsc1*-cKO mice retina. Cryosections of the 4- and 6-week-old *Tsc1*-cKO and age-matched control mice retina were stained with GFAP and Iba1. GFAP staining was faint in 4-week-old *Tsc1*-cKO mice retina. It became strong in the 6-week-old *Tsc1*-cKO mice retina. Similarly, Iba1 staining was faint 4-week-old *Tsc1*-cKO mice retina, and became strong in 6-week-old retina. The results suggested that Müller glial and microglial cells of the *Tsc1*-cKO retina were activated at around 6-week of age. Scale bar: 100 μm. **(D).** Relative cell soma size of PKCα-positive bipolar cells in Tsc1-cKO retina compared to controls. Cell soma size was calculated on an immunofluorescent stained retina section using ImageJ. A retinal section of 500 μm in length was chosen and the total area of positively stained cell soma at the INL was totaled. Three staining images from 3 different animals were used and the data was averaged. Error bars represent standard error and * represents a p-value of less than 0.05. The stainings of the 3-month-old retina sections were used for both control and Tsc1-ckO mice.

**Supplementary Figure S3**

**
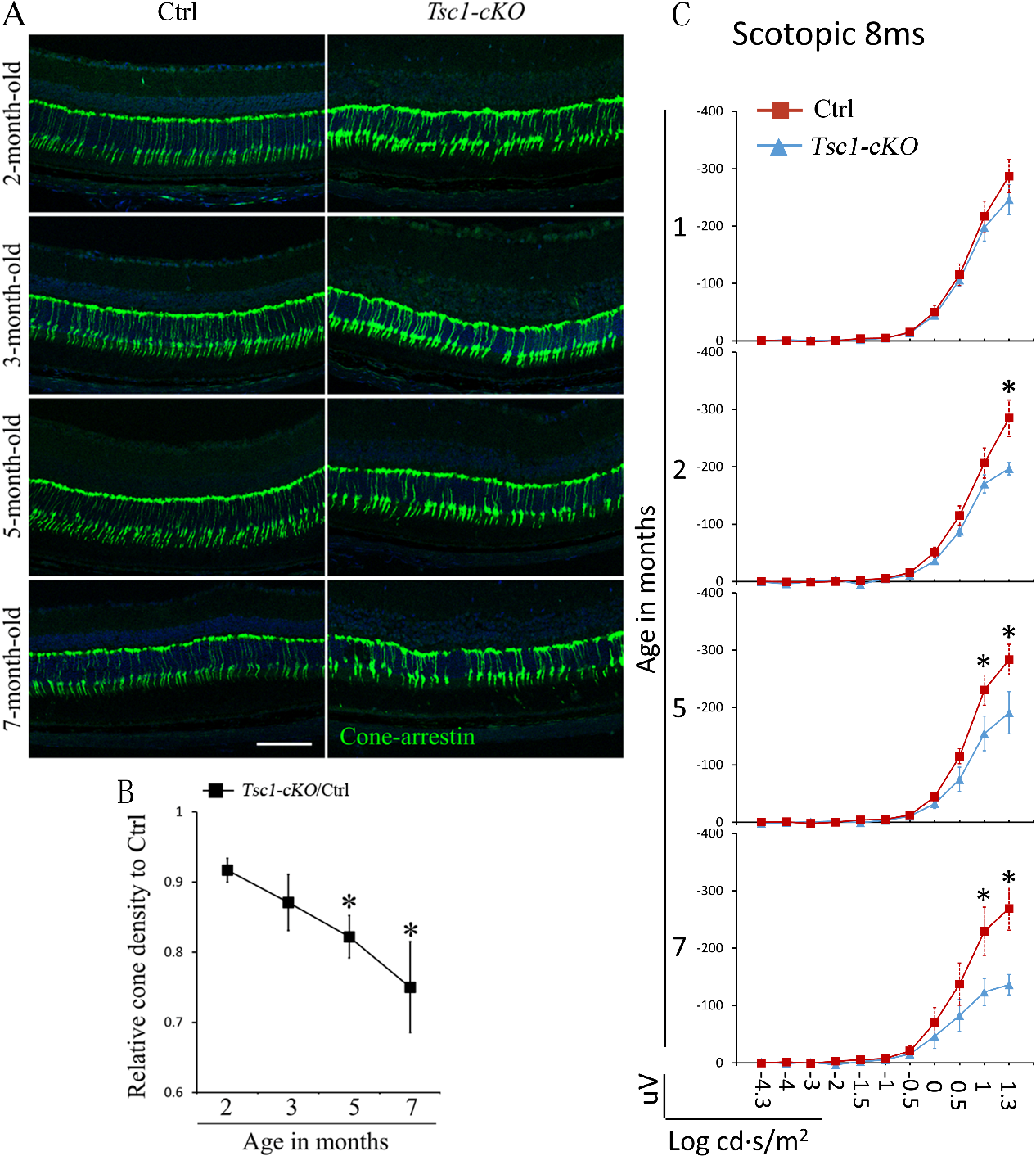
**

**Supplementary Figure S3. Progressive loss of cone photoreceptor cells and the associated decrease in ERG responses in *Tsc1*-cKO mice.** **(A).** Representative Immunofluorescent staining of cone-arrestin on retina samples taken from 2-, 3-, 5- and 7-month-old *Tsc1*-cKO and age-matched control mice. Scale bar: 100 μm. **(B**). Quantitative analysis of cone cell density in Tsc1-cKO retina compared to controls. The number of arrestin-positive cells per image of 500 µm in length was counted and the number of the control retina at each age was set at 100% and used to calculate the relative percentage of cones in the knockout retina. Stainings of 3 different retina in each group of each age were counted and data was averaged. Error bars represent standard error and * represents a p-value of less than 0.05. **(C).** Amplitudes of ERG at 8 ms after the flash under scotopic conditions in Tsc1-cKO and control mice of different ages. Data from 6 mice from each age group of each genotype were averaged. Error bars represent standard error and * represents a p-value of less than 0.05.

**Supplementary Table S3.**

GO terms significantly upregulated in 5-month-old *Tsc1*-cKO retina compared to age-matched controls.

| Term description | ListHit | FDR | Enrichment score |
| --- | --- | --- | --- |
| Biological Process |  |  |  |
| **Immune system process (GO:0002376)** | 142 | 3.68E-17 | 2.17 |
| Defense response to virus (GO:0002376) | 63 | 8.35E-10 | 2.49 |
| Innate immune response (GO:0045087) | 121 | 8.35E-10 | 1.89 |
| Positive regulation of angiogenesis (GO:0045087) | 50 | 1.45E-06 | 2.35 |
| Response to virus (GO:0009615) | 26 | 4.35E-06 | 3.25 |
| Immune response (GO:0006955) | 68 | 4.77E-06 | 1.99 |
| Negative regulation of viral genome replication (GO:0045071) | 22 | 8.66E-06 | 3.48 |
| Inflammatory response (GO:0006954) | 90 | 6.37E-05 | 1.70 |
| Chemotaxis (GO:0006935) | 45 | 9.34E-05 | 2.15 |
| Response to interferon-gamma (GO:0034341) | 15 | 1.74E-04 | 3.90 |
| Regulation of apoptotic process (GO:0042981) | 74 | 1.79E-04 | 1.75 |
| **Regulation of cell proliferation (GO:0042127)** | 66 | 1.24E-03 | 1.72 |
| Positive regulation of apoptotic process (GO:0043065) | 84 | 1.24E-03 | 1.61 |
| Cellular response to interferon-beta (GO:0035458) | 18 | 1.52E-03 | 3.00 |
| Regulation of blood pressure (GO:0008217) | 23 | 3.48E-03 | 2.49 |
| Neutrophil chemotaxis (GO:0030593) | 23 | 5.64E-03 | 2.41 |
| Cellular response to interferon-gamma (GO:0071346) | 29 | 5.64E-03 | 2.16 |
| Cell surface receptor signaling pathway (GO:0007166) | 53 | 5.64E-03 | 1.74 |
| Wound healing (GO:0042060) | 25 | 6.29E-03 | 2.29 |
| Positive regulation of ERK1 and ERK2 cascade (GO:0070374) | 61 | 6.29E-03 | 1.66 |
| Response to interferon-beta (GO:0035456) | 8 | 6.31E-03 | 4.72 |
| Antigen processing and presentation of peptide antigen via MHC class I (GO:0002474) | 14 | 9.26E-03 | 3.03 |
| Positive regulation of fibroblast proliferation (GO:0048146) | 23 | 9.67E-03 | 2.30 |
| Intracellular signal transduction (GO:0035556) | 100 | 9.67E-03 | 1.45 |
| Cellular Component |  |  |  |
| **Extracellular region (GO:0005576)** | 379 | 6.62E-14 | 1.48 |
| **Extracellular space (GO:0005615)** | 325 | 7.42E-12 | 1.48 |
| Cytoplasm (GO:0005615) | 1189 | 7.5E-12 | 1.18 |
| **Plasma membrane (GO:0005886)** | 748 | 1.5E-10 | 1.24 |
| **Membrane (GO:0016020)** | 1147 | 9.29E-09 | 1.16 |
| Extracellular matrix (GO:0031012) | 70 | 2.01E-08 | 1.00 |
| Proteinaceous extracellular matrix (GO:0005578) | 90 | 4.75E-07 | 1.82 |
| Cytoskeleton (GO:0005856) | 250 | 6.51E-06 | 1.36 |
| **Cell surface (GO:0009986)** | 146 | 1.52E-05 | 1.50 |
| **External side of plasma membrane (GO:0009897)** | 82 | 8.59E-05 | 1.67 |
| Collagen trimer (GO:0005581) | 27 | 5.95E-04 | 2.34 |
| Actin cytoskeleton (GO:0015629) | 535 | 8.03E-04 | 1.17 |
| Cytosol (GO:0005829) | 55 | 8.03E-04 | 1.75 |
| **MHC class I protein complex (GO:0042612)** | 8 | 1.67E-03 | 4.73 |
| Symbiont-containing vacuole membrane (GO:0020005) | 7 | 2.57E-03 | 5.06 |
| Cell junction (GO:0030054) | 158 | 2.69E-03 | 1.33 |
| Basement membrane (GO:0005604) | 29 | 9.02E-03 | 1.94 |
| Molecular Function |  |  |  |
| Protein binding (GO:0005515) | 895 | 5.85E-09 | 1.20 |
| Metal ion binding (GO:0046872) | 555 | 1.47E-04 | 1.20 |
| Identical protein binding (GO:0042802) | 241 | 1.47E-04 | 1.35 |
| **Calcium ion binding (GO:0005509)** | 137 | 3.45E-04 | 1.47 |
| Actin binding (GO:0003779) | 87 | 4.07E-04 | 1.63 |
| Protein homodimerization activity (GO:0042803) | 189 | 4.87E-04 | 1.37 |
| Zinc ion binding (GO:0008270) | 141 | 2.59E-03 | 1.40 |
| Double-stranded RNA binding (GO:0003725) | 25 | 3.53E-03 | 2.32 |

GO enrichment analysis of significantly upregulated genes in 5-month-old *Tsc1*-cKO mice compared to age-matched control mice were performed using R based on the hypergeometric distribution. The significantly enriched GO terms (FDR<0.01) were listed above. The terms in bold are those which were also upregulated in 24-month-old C57BL/6 mice group.
